# The CARD8 T60 variant associates with NLRP1 and negatively regulates its activation

**DOI:** 10.3389/fimmu.2022.1047922

**Published:** 2022-11-08

**Authors:** Zhihao Xu, Shasha Deng, Yuluo Huang, Yunru Yang, Liangqi Sun, Hanyuan Liu, Dan Zhao, Weihong Zeng, Xueying Yin, Peiyi Zheng, Yingying Wang, Muziying Liu, Weidong Zhao, Tsan Sam Xiao, Ying Zhou, Tengchuan Jin

**Affiliations:** ^1^ Department of Obstetrics and Gynecology, Core Facility Center, The First Affiliated Hospital of USTC, Division of Life Sciences and Medicine, University of Science and Technology of China, Hefei, Anhui, China; ^2^ Laboratory of Structural Immunology, the CAS Key Laboratory of Innate Immunity and Chronic Disease, School of Basic Medicine Sciences, Division of Life Sciences and Medicine, University of Science and Technology of China, Hefei, Anhui, China; ^3^ Anhui Institute of Pediatric Research, Anhui Provincial Children’s Hospital, Hefei, China; ^4^ Department of Pathology, Case Western Reserve University, Cleveland, OH, United States; ^5^ CAS Center for Excellence in Molecular Cell Science, Chinese Academy of Science, Shanghai, China

**Keywords:** NLRP1 inflammasome, CARD8, death domain superfamily, CARD, autoinflammatory diseases

## Abstract

The NLRP1 inflammasome functions as canonical cytosolic sensor in response to intracellular infections and is implicated in auto-inflammatory diseases. But the regulation and signal transduction mechanisms of NLRP1 are incompletely understood. Here, we show that the T60 variant of CARD8, but not the canonical T48 isoform, negatively regulates the NLRP1 inflammasome activation by directly interacting with the receptor molecule NLRP1 and inhibiting inflammasome assembly. Furthermore, our results suggest that different ASC preference in three types of inflammasomes, namely the ASC-indispensable NLRP1 inflammasome, ASC-dispensable mNLRP1b inflammasome and ASC-independent CARD8 inflammasome, is mainly caused by the CARD domain, not the UPA subdomain. Based on the systematic site-directed mutagenesis and structural analysis, we find that signal transduction of the NLRP1 inflammasome relies on multiple interaction surfaces at its CARD domain. Finally, our results partly explain how mutations in NLRP1 lead to its constitutive activation in auto-inflammatory diseases. In conclusion, our study not only reveals how CARD8 downregulates the NLRP1 inflammasome activation, but also provides insights into the assembly mechanisms of CARD-containing inflammasomes.

## Introduction

The canonical inflammasomes are cytosolic multiprotein complexes that initiate pyroptotic cell death and inflammatory cytokine secretion in response to diverse pathogenic and endogenous danger signals (1). NLRP1, the first discovered inflammasome sensor, could assemble inflammasome macromolecular platform by oligomerizing with ASC (also known as PYCARD) and recruiting the effector inflammatory molecule proCaspase-1 (2). It facilitates the processing of pro-interleukin-1β (proIL-1β), pro-interleukin-18 (proIL-18) and gasdermin D (GSDMD) that results in the release of mature cytokines and induction of inflammatory cell death. Recently, some specific pathogen-associated molecular patterns (PAMPs), such as enteroviral 3C proteases and the double-stranded RNA, were identified that can activate the human NLRP1 inflammasome (3, 4). In addition, the dipeptidyl peptidases DPP8/9 can directly interact with FIINDs domain of human NLRP1 and CARD8 (5–7). Consistently, DPP8/9 inhibitors, such as Val-boroPro (VbP), induce NLRP1 and CARD8 inflammasome assembly through their UPA-CARD domains generated upon proteasome-mediated degradation of their respective N-terminal fragments (8–11).

In addition, mutations in NLRP1 result in constitutive NLRP1 inflammasome overactivation and inflammatory cytokines secretion, which have been associated with a number of chronic inflammatory disorders, including autoinflammation with arthritis and dyskeratosis (AIADK) and familial keratosis lichenoides chronica (FKLC) (12, 13). The NLRP1 inflammasome is maintained in an inactive state to prevent aberrant activation and damaging inflammation in the absence of infection and danger related molecules. Thus, understanding the negative regulation mechanism of the NLRP1 inflammasome is critical for discovery of novel therapy strategies for these auto-inflammatory diseases.

NLRP1 and CARD8 share similar domain organization, including the C-terminal function-to-find domain (FIIND) and caspase activation and recruitment domain (CARD). The FIIND domain has a proteolytic site that splits it into ZU5 (found in ZO-1 and UNC5) and UPA (conserved in UNC5, PIDD, and ankyrins) subdomains that associate noncovalently to assemble the auto-inhibited N-terminal fragments. The CARD is a member of the death domain (DD) superfamily that engages in the assembly of inflammasome complexes through homotypic and heterotypic interactions. The CARD containing ASC plays a significant role in mediating the interactions between sensors and the cysteine proteases caspase-1. In contrast to human CARD8, which directly interacts caspase-1 and instead ASC engages (14), ASC functions as an indispensable adaptor to activate caspase-1 in human NLRP1 inflammasome (15). Additionally, human NLRC4 and murine NLRP1b can form a functional complex both with ASC and without ASC (16–18). The specificity of NLRP1 and CARD8 for ASC is obviously different for most inflammasome sensors because of poorly defined UPA subdomain. The detailed analysis of how CARD-containing sensor proteins recruit downstream molecules has not been reported.

Interestingly, CARD8 has been reported to function as a negative regulator of NLRs in human, but not in rodents. Some investigators showed that CARD8 binds to NOD2 and suppresses NOD2-dependent inflammatory signal transduction (19). Recent publications report that CARD8 interacts physically with NLRP3 to prevent its binding to the adaptor protein ASC during the NLRP3 inflammasome activation (20, 21). Since NLRP1 and NLRP3 share similar domain structures, we investigated potential roles of CARD8 in regulating the NLRP1 inflammasome. In this study, we characterize CARD8 as a negative regulator of the NLRP1 inflammasome activation through its direct interaction with NLRP1, and provide the molecular basis for the specificity of CARD-mediated inflammasomes assembly.

## Results

### CARD8-T48 isoform interacts with NLRP1 but fails to inhibit its functions

**Figure 1:**
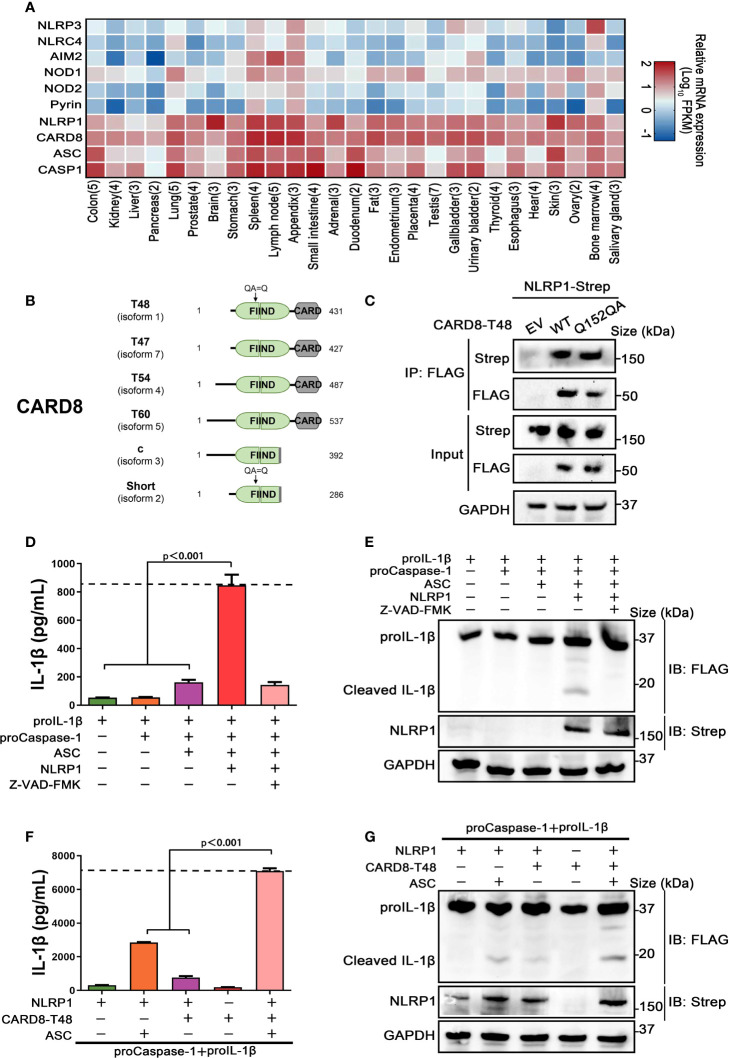
CARD8-T48 isoform interacts with NLRP1, but it does not inhibit NLRP1 activation. **(A)** Heat map of NLRP1 and other inflammasome sensors transcription expression levels in 27 kinds of tissues or organs in human body. The numbers of replicate samples are labeled in parentheses. **(B)** Diagram of human CARD8 different isoforms. Full-length CARD8 protein consists of a variable aminoterminal disordered region, FIIND and CARD domain. **(C)** HEK 293T cells were transiently transfected with constructs for empty vector (EV), CARD8-T48-FLAG, CARD8-T48-Q152QA-FLAG and NLRP1-Strep. After 28 hours, the protein expression was detected by immunoblotting, and co-immunoprecipitation (IP) were conducted with anti-FLAG antibody to analyze the interaction. **(D, E)** HEK 293T cells were transiently transfected with constructs encoding the proIL-1β, proCaspase-1, ASC and NLRP1. At 48 hours later, the cultural supernatants were monitored for cleaved IL-1β by ELISA and cell lysates were evaluated by immunoblotting. Data are shown as mean ± SEM and representative of three biological replicates. **(F, G)** HEK 293T cells were transiently transfected with constructs encoding the proIL-1β, proCaspase-1, ASC, NLRP1 and CARD8-T48. After 48 hours, ELISA and immunoblotting analysis were conducted with cell cultural supernatants and lysates. Data are shown as mean ± SEM and representative of three biological replicates.

Previous studies have demonstrated that CARD8 is not only an inflammasome-forming sensor, but also acts as a negative regulator in several pro-inflammatory and apoptotic signaling pathways (21–23). To further evaluate the roles of CARD8 in regulating the NOD-like receptors family, protein-protein interaction network was performed with an analysis tool STRING (24). Despite NLRP1 and CARD8 having similar domain structures, the molecular association of CARD8 and NLRP1 remains to be clarified and verified experimentally (**Figure S1**). Then, we examined the tissue expression profile of multiple inflammasome components using available transcriptome data from 27 different human organs and tissues (25). Based on specimens from 95 individuals, we found that CARD8 and NLRP1 are widely expressed, in contrast to other known inflammasome receptors (**Figure 1A**). In addition, CARD8 possesses a similar tissue expression profile as compared to NLRP1, perhaps indicating that CARD8 and NLRP1 have a functional molecular association. Based on these observations, the analysis of the CARD8 polymorphism was performed to investigate the regulatory role of CARD8 in inflammasome signal transduction pathways. CARD8 mainly contains six different isoforms, among which the T48 isoform was considered as canonical sequence previously (**Figure 1B**) (26). Compared with the T48 isoform, the T60 isoform exhibited a longer unstructured N-terminal region and an additional alanine in the ZU5 subdomain. This may suggest that distinct isoforms of CARD8 play different roles in mediating signal transduction. To investigate the interaction between the wild-type full-length CARD8 and NLRP1, co-immunoprecipitation assay was performed. The result showed that the CARD8-T48 isoform was immunoprecipitated with NLRP1, suggesting that CARD8-T48 isoform can bind to NLRP1 in HEK 293T cells (**Figure 1C**). To ascertain whether CARD8 could affect the NLRP1 inflammasome activation, we co-transfected NLRP1 inflammasome components to reconstitute the NLRP1 inflammasome in HEK 293T cells. The co-expression of NLRP1, ASC, proCaspase-1 and proIL-1β can result in the caspase-1-mediated IL-1β maturation and secretion, showing that the NLRP1 reconstitution system could be used to study the NLRP1 inflammasome regulation mechanism (**Figures 1D, E**). Then, the CARD8-T48 isoform was introduced into HEK 293T cells to determine the regulatory function of CARD8 using the reconstitution system. We found that CARD8-T48 isoform did not cause a defect in processing of proIL-1β by Caspase-1, indicating no inhibitory function of CARD8-T48 on NLRP1 inflammasome activation in HEK 293T cells (**Figures 1F, G**). Taken together, these results showed that CARD8-T48 isoform binds with NLRP1 but fails to inhibit NLRP1 inflammasome activation.

### Negative regulation of NLRP1 inflammasome activation level by CARD8-T60 isoform

**Figure 2:**
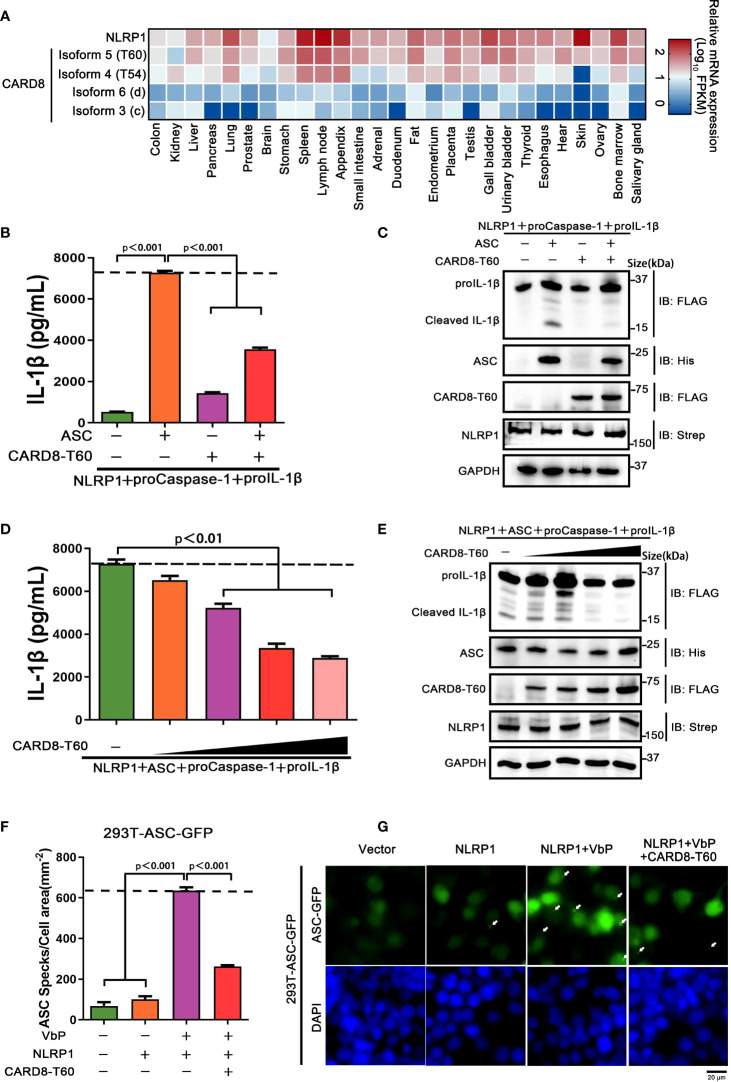
CARD8-T60 isoform suppresses NLRP1 inflammasome activation level. **(A)** Heat map of NLRP1 and CARD8 isoforms transcription expression levels in 27 kinds of tissues or organs in human body. **(B, C)** HEK 293T cells were transiently transfected with constructs encoding the proIL-1β, proCaspase-1, ASC, NLRP1 and CARD8-T60. After 48 hours, ELISA and immunoblotting analysis were conducted with cell cultural supernatants and lysates. Data are shown as mean ± SEM and representative of three biological replicates. **(D, E)** HEK 293T cells were transiently transfected with proIL-1β, proCaspase-1, ASC, NLRP1 and an increased concentration CARD8-T60 expression plasmids. After 48 hours, ELISA and immunoblotting analysis were conducted with cell cultural supernatants and lysates. Data are shown as mean ± SEM and representative of three biological replicates. **(F, G)** HEK 293T cells stably expressing ASC-GFP were transiently transfected with the indicated expression plasmids and treated with VbP (5 μM) for 6 hours. The cells were fixed with 4% formaldehyde and evaluated for ASC speck formation by fluorescence microscopy in three differential regions. Data of mean specks per cell area are shown as mean ± SEM in f and representative images are shown in g.

As noted above, the CARD8 has six different isoforms and similar tissue distributions with NLRP1. To identify whether other different CARD8 isoforms could affect the NLRP1 inflammasome activation, we first characterized the expression levels of distinct CARD8 isoforms in 27 different human organs and tissues. To our surprise, CARD8-T60 isoform exhibited a much higher expression level compared with other CARD8 isoforms (**Figure 2A**). To investigate the effect of CARD8-T60 isoform in NLRP1 inflammasome, CARD8-T60 isoform was expressed, which led to significantly decreased cleavage products of proIL-1β (**Figures 2B, C**). To further examine the influence of CARD8-T60 on NLRP1-mediated activation, the maturation and secretion level of IL-1β upon expression of different amount of CARD8-T60 was measured. In the reconstituted NLRP1 inflammasome system, the secretion level of Caspase-1-mediated release of IL-1β was considerably reduced in a dose-dependent manner (**Figures 2D, E**).

We sought to investigate the molecular mechanism of the CARD8-T60 isoform on NLRP1 inflammasome activation. As inflammasome signaling platform assembly is essential for NLRP1 inflammasome activation, we next corroborated the role of CARD8-T60 in the assembly of the NLRP1 inflammasome. HEK 293T cells were stably expressed with ASC-GFP fluorescent fusion protein to examine the number of ASC speck-like aggregates (**Figure S2**). Through inspecting ASC-speck formation in 293T-ASC-GFP cells, we found that NLRP1 inflammasome assembly was significantly diminished in the presence of the full-length CARD8-T60 isoform (**Figure 2F, G**). Altogether, these findings showed that the CARD8-T60 isoform could suppress the formation and activation of NLRP1 inflammasome in a dose-dependent manner.

### CARD8-T60 directly interacts with NLRP1

**Figure 3:**
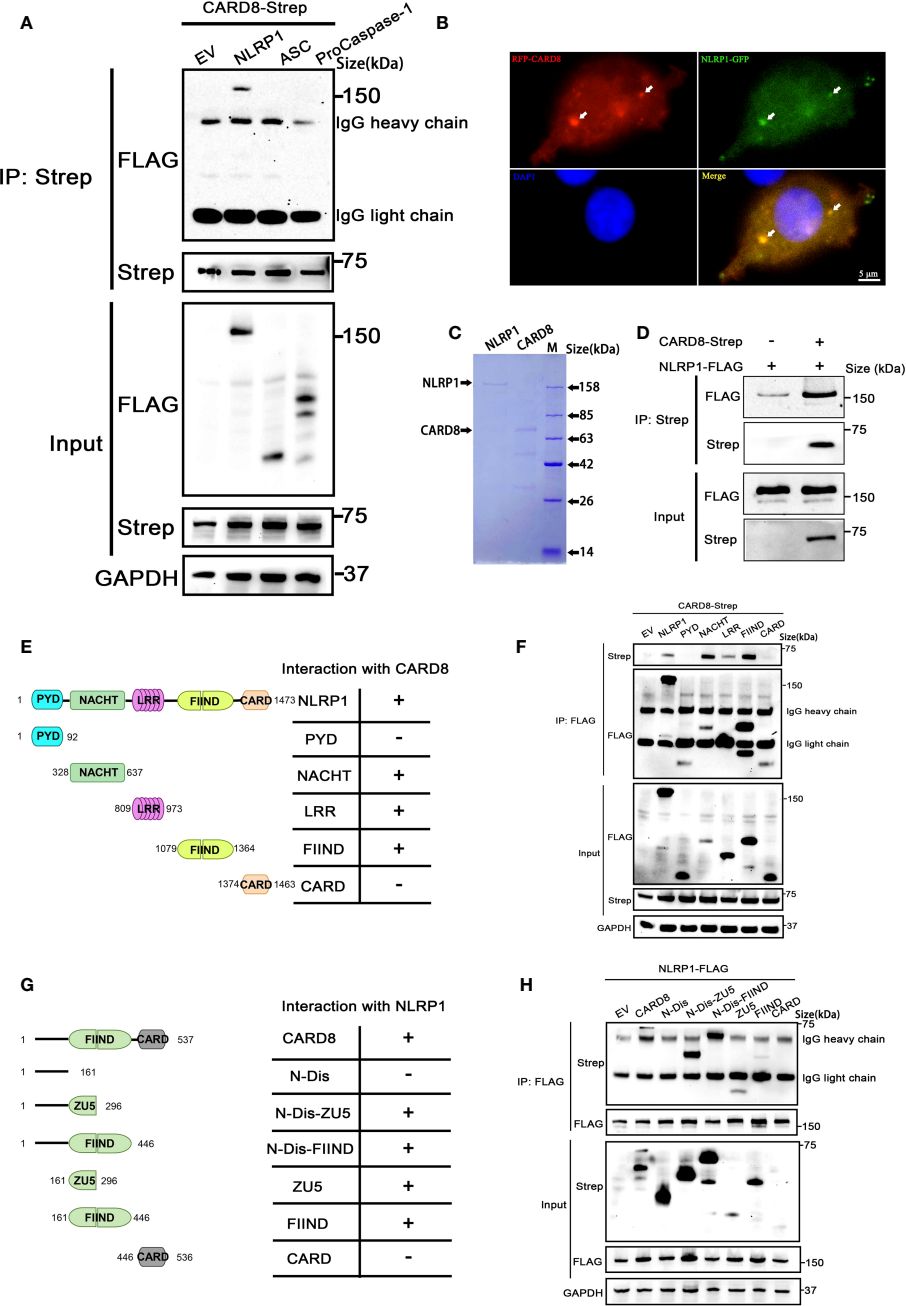
CARD8-T60 co-localizes and directly interacts with NLRP1. **(A)** HEK 293T cells were transiently transfected with constructs for empty vector (EV), NLRP1-FLAG, ASC-FLAG, proCaspase-1-FLAG and CARD8-T60-Strep. After 28 hours, the protein expression was detected by immunoblotting, and co-immunoprecipitation were conducted with anti-Strep antibody to analyze the interaction. **(B)** Transient transfection RFP-CARD8 and NLRP1-GFP in HEK 293T cells. Green and red fluorescent were captured by fluorescence microscopy. Co-localization regions are labeled with white arrow. **(C)** The SDS-PAGE analysis of recombinant NLRP1 and CARD8 proteins. **(D)** Pull down assay of purified NLRP1-FLAG and CARD8-Strep proteins *in vitro*. **(E)** Diagrammatic representation of truncation mutants of NLRP1. **(F)** HEK 293T cells were transiently transfected with constructs for empty vector (EV), NLRP1-FLAG, NLRP1-FLAG truncation mutants and CARD8-T60-Strep. After 28 hours, the protein expression was detected by immunoblotting, and co-immunoprecipitation were conducted with anti-FLAG antibody to analyze the interaction. **(G)** Diagrammatic representation of truncation mutants of CARD8-T60. **(H)** HEK 293T cells were transiently transfected with constructs for empty vector (EV), CARD8-T60-Strep, CARD8-T60-Strep truncation mutants and NLRP1-FLAG. After 28 hours, the protein expression was detected by immunoblotting, and co-immunoprecipitation were conducted with anti-FLAG antibody to analyze the interaction.

In order to clarify the molecular mechanism of how to CARD8-T60 isoform inhibits NLRP1 inflammasome activation, we next analyzed the interaction between CARD8-T60 and components of the NLRP1 inflammasome. Co-immunoprecipitation revealed the association between CARD8-T60 and full-length NLRP1, but not ASC or proCaspase-1 (**Figure 3A**). With the co-expression of RFP-CARD8 and NLRP1-GFP, the fluorescent signal showed significant co-localization of CARD8 and NLRP1 in HEK 293T cells (**Figure 3B**). The similar cytoplasmic distribution of CARD8 and NLRP1 suggests that CARD8 and NLRP1 interact with each other in cells. Furthermore, we observed that RFP-CARD8 was also colocalized with the murine mNLRP1b with a high colocalization coefficient similar to the human NLRP1, suggesting that CARD8-NLRP1 interaction is an evolutionarily conserved event (**Figures S3A, B**). To investigate whether the association of CARD8 and NLRP1 is through direct interaction, we evaluated the interaction of CARD8 and NLRP1 using purified recombinant CARD8 and NLRP1 proteins *in vitro* (**Figure S3C**). Soluble Flag-tagged NLRP1 and CARD8 proteins were purified using mammalian protein expression system (**Figure 3C**). Further co-immunoprecipitation assay showed that NLRP1 was pulled down by CARD8 which suggests their direct interaction (**Figure 3D**).

Moreover, to ascertain the details of the CARD8-NLRP1 interaction, we conducted a systematic mapping analysis of the binding of CARD8 with NLRP1. The co-immunoprecipitation experiments were performed using different domains of NLRP1 with the full-length CARD8 and vice versa. This analysis revealed that the CARD8-binding domains in NLRP1 are the NACHT, LRR and FIIND domains (**Figures 3E, F**). Similarly, we found that the CARD8-NLRP1 interaction required the minimal ZU5 subdomain of CARD8 (**Figures 3G, H**). Comparing the domain structure of the T48 and T60 isoforms, T60 has a longer N-terminal disordered region and an additional alanine at the ZU5 subdomain. It seems that this difference results in a completely different regulation on the NLRP1 inflammasome. The presence of the N-terminal unstructured region of the CARD8-T60 isoform could increase the binding capability between NLRP1 and CARD8 (**Figure 3H**), but the mutant of CARD8-T48 isoform with an alanine inserted in its ZU5 subdomain (Q152QA) has no significant change in the CARD8-T48 and NLRP1 interaction (**Figure 1C**). These results demonstrated once again that CARD8 directly binds NLRP1 to inhibit its activation level and there is a constitutive interaction of CARD8 with NLRP1 where the ZU5 (CARD8) subdomain binds to the NACHT, LRR, FIIND domain of NLRP1.

### Distinct ASC preference is dependent on CARD, but not UPA domain, in NLRP1, mNLRP1b and CARD8 inflammasome

During the investigation into the effect of CARD8 on NLRP1 inflammasome, the data show that human CARD8 could induce the secretion of IL-1β in the absence of ASC, while NLRP1 does not (**Figures 1F**, **2B**). The inflammasome assembly is extremely complicated and the assembly mechanism of CARD-containing inflammasomes remains elusive, including mouse mNLRP1b, human NLRP1 and CARD8 inflammasome. We aim to systematically analyze the ASC preference in these CARD-containing inflammasomes. The adaptor protein ASC is necessary for IL-1β secretion in human NLRP1 inflammasome, while no significant differences in IL-1β secretion were observed in the presence or absence of ASC for human CARD8 inflammasome (**Figures S4A, B**). For murine mNLRP1b inflammasome, it can directly activate downstream signal pathways with or without ASC bridging, but the participation of ASC strongly promotes the activation level of mNLRP1b inflammasome (**Figures S4A, B**). Moreover, the ASC-containing inflammasome assembly can be detected in 293T-ASC-GFP cells with mNLRP1b and NLRP1 expression, but not in CARD8-T48 and CARD8-T60 expressing cells (**Figures S4C, D**). Our results are consistent with previous reports (14, 15, 27, 28) that ASC is an indispensable, dispensable and independent adaptor in NLRP1, mNLRP1b and CARD8 inflammasome assembly, respectively.

**Figure 4:**
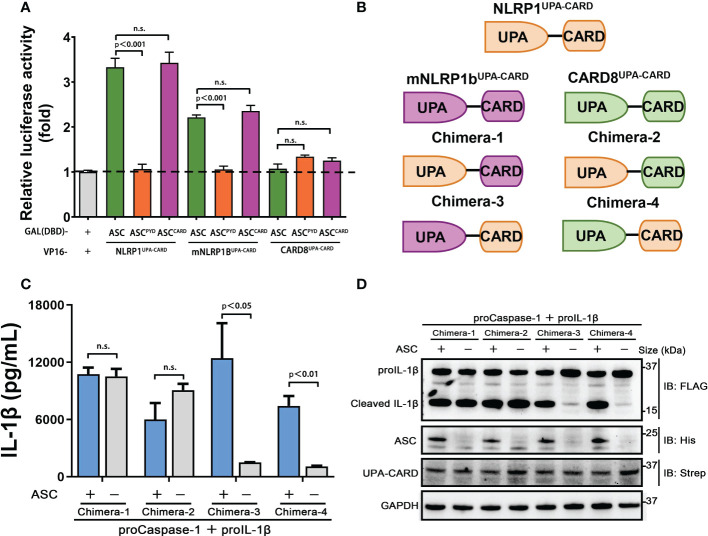
CARDs play the key roles for ASC dependence in NLRP1 and CARD8 inflammasome. **(A)** Mammalian two-hybrid characterization for the interaction of ASC with NLRP1^UPA-CARD^, mNLRP1b^UPA-CARD^ and CARD8^UPA-CARD^. Luciferase activity was normalized to *Renilla* and data were shown as the fold of empty vector control. Mean values ± SEM are representative of three biological replicates. **(B)** Diagram of the chimera proteins of NLRP1^UPA-CARD^, mNLRP1b^UPA-CARD^ and CARD8^UPA-CARD^. **(C,D)** ELISA and immunoblotting analysis were conducted from ASC-deficient chimera inflammasome. Data are shown as mean ± SEM and representative of three biological replicates. n.s., not significant.

The C-terminal structural segment containing a UPA subdomain and an entire CARD domain is responsible for recruiting downstream signal molecules to complete the inflammasome assembly when NLRP1, mNLRP1b and CARD8 are activated by danger signals (29). We next examined whether the distinct ASC preference for NLRP1, mNLRP1b and CARD8 inflammasome depends on UPA subdomain or CARD domain. We first employed the mammalian two-hybrid (M2H) system to assess the interaction of NLRP1, mNLRP1b and CARD8 with ASC. The UPA-CARD fragment of NLRP1 and mNLRP1b, but not CARD8, showed a strong binding with ASC in agreement with results from the functional studies (**Figure 4A**). In addition, the M2H results also showed that the homotypic CARD-CARD interaction plays an important role in mediating the interaction of NLRP1 and mNLRP1b with ASC (**Figure 4A**). Then, we generated a series of chimeric proteins for the UPA-CARD domain of NLRP1, mNLRP1b and CARD8 (**Figure 4B**). Chimera-1 and chimera-2, in which the NLRP1^CARD^ domain was substituted with the CARD domain of mNLRP1b or CARD8, could activate the inflammasome-mediated signals independent of ASC (**Figures 4C, D**). Meanwhile, replacing the mNLRP1b^CARD^ or CARD8^CARD^ domain with CARD domain of NLRP1 (chimera-3 and chimera-4, respectively) blocked the proIL-1β maturation and secretion in the absence of ASC (**Figures 4C, D**). By the analysis of the ASC-dependent sensor proteins, all NLRP1^UPA-CARD^, chimera-3 and chimera-4 share the common structural feature including the same CARD domain of human NLRP1. We conclude that the distinct ASC preference depends on the CARD domain in NLRP1, mNLRP1b and CARD8 inflammasome, while the UPA subdomain does not determine the specificity to assemble distinct inflammasome complexes.

### Structure basis for CARD domain-mediated ASC preference

**Figure 5:**
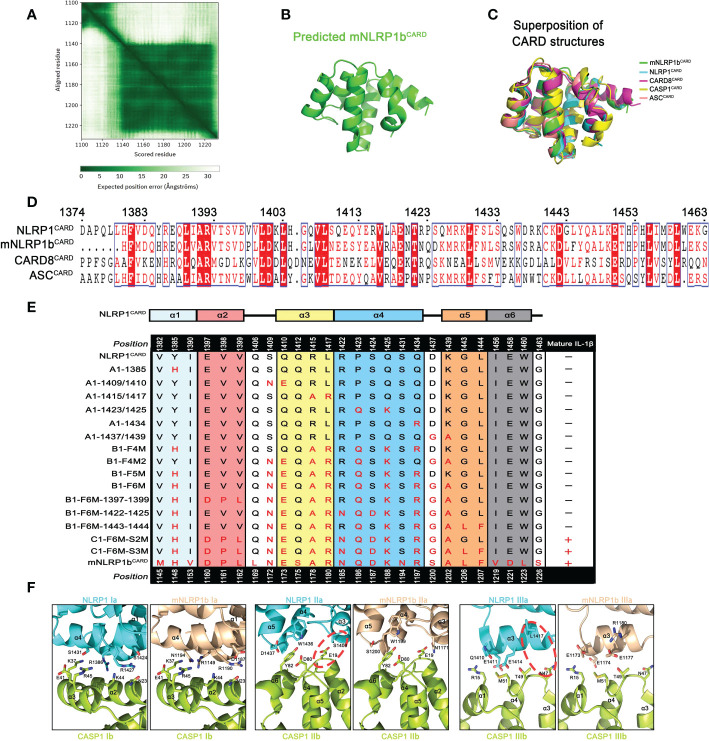
Various CARD interfaces were critical for ASC dependence in NLRP1 and CARD8 inflammasome. **(A)** The AlphaFold’s predicted aligned error of mNLRP1b encoding residues 1100-1233 structure. **(B)** Predicted structure of the CARD domain of mNLRP1b. **(C)** Superposition of all five known CARDs from mNLRP1b (green), NLRP1 (cyan), CARD8 (magenta), CASP1 (yellow), and ASC (lightpink). **(D)** Sequence alignment analysis of hNLRP1^CARD^, mNLRP1b^CARD^, CARD8^CARD^ and hASC^CARD^. Amino acid position is shown on the top of the sequences. **(E)** Mapping critical hNLRP1^CARD^ interaction sites for ASC-deficient NLRP1 inflammasome activation based on mNLRP1b^CARD^ sequence. The secondary structure of hNLRP1^CARD^ is indicated on the top. **(F)** Structural compatibility for Type I, II, and III interaction residues of CASP1^CARD^ with human NLRP1^CARD^ or murine mNLRP1b^CARD^. Red line is used to label possible clashes in detailed CARD-CARD interaction model.

Based on the above results, the CARD domain is a key determinant in the ASC dependence for the assembly of the murine mNLRP1b, human NLRP1 and CARD8 inflammasomes. We next performed structural analysis focusing on the CARD domain. The crystallographic structures of the C-terminal CARD domains of NLRP1 and CARD8 were reported in prior studies (30, 31), but the three-dimensional structure of the mNLRP1b^CARD^ remains unknown. The predicted structure of mNLRP1b^CARD^ by AlphaFold (32, 33) has a high confidence because of the conserved three-dimensional structures of the Death Domain superfamily members (**Figure 5A**). The C-terminal CARD domain of the predicted mNLRP1b structure is composed of six anti-parallel α-helices folded in a Greek key arrangement (**Figure 5B**). The five DD superfamily members, including mNLRP1b^CARD^, NLRP1^CARD^ (4IFP), CARD8^CARD^ (4IKM), CASP1^CARD^ (5FNA), ASC^CARD^ (6KI0), can be aligned with rmsds between 0.6 Å and 2.2 Å, which confirms the conserved structural characteristics of the CARD domains (**Figure 5C**).

Since the structures of mNLRP1b^CARD^, NLRP1^CARD^ and CARD8^CARD^ showed a high similarity, we further determined the contribution of different amino acids in the CARD domain that may result in the distinct ASC preference. Sequence alignment of NLRP1^CARD^, mNLRP1b^CARD^, CARD8^CARD^ and ASC^CARD^ suggested a low sequence identity of CARD domains (**Figure 5D**). Thus, we analyzed how sequence differences in the CARD domains contribute to their ASC dependence for the inflammasome assembly. Site-directed mutagenesis on NLRP1^UPA-CARD^ was conducted to map critical surface sites that may mediate the interaction between NLRP1^UPA-CARD^ and downstream signal molecules according to the primary sequence of mNLRP1b^UPA-CARD^ (**Table S1**). The systematic site-directed mutagenesis analysis confirmed that the sixth α-helix of NLRP1^CARD^ does not affect NLRP1 inflammasome assembly to carry out signal amplification. More importantly, we also found that multiple molecular surfaces were involved in the interaction of NLRP1^UPA-CARD^ and Caspase-1^CARD^ to transduce downstream signals, which is consistent with the Mosaic model that DD superfamily members co-assemble into archetypical supramolecular platform by three conserved interaction types (15) (**Figure 5E** and **Figure S5**).

Type I, Type II, and Type III interaction types of DD superfamily members, first defined in MyDDosome complex assembly, were widely adopted in describing supramolecular filament formations of DD superfamily (34). On the other hand, the structural conformations of NLRP1^CARD^ and mNLRP1b^CARD^ are similar to the structure of ASC^CARD^ with rmsds 0.814 Å and 0.718 Å, respectively. To elucidate the heterotypic interactions between CARDs of sensors and downstream effector Caspase-1^CARD^, we modeled three asymmetric interfaces of DD superfamily between NLRP1^CARD^ or mNLRP1b^CARD^ and Caspase-1^CARD^ based on the cryo-EM structure of octamer with 4 ASC^CARD^ molecules and 4 Caspase-1^CARD^ molecules (PDB: 7KEU) (35). As shown in **Figure 5F**, the interaction of mNLRP1b^CARD^ and Caspase-1^CARD^ is favored, whereas NLRP1^CARD^ clashes with Caspase-1^CARD^. For example, the E19 and N47 on the Caspase-1^CARD^ Type IIb and IIIb surfaces show strong compatibility with corresponding positions N1171 and R1180 in mNLRP1b^CARD^, but not the S1409 and L1417 on the NLRP1^CARD^ Type IIa and IIIa surfaces. Taken together, our results provide a detailed structural foundation for ASC preference during most CARD-containing inflammasome assembly.

### The functional implications of patient-derived NLRP1 mutations

**Figure 6:**
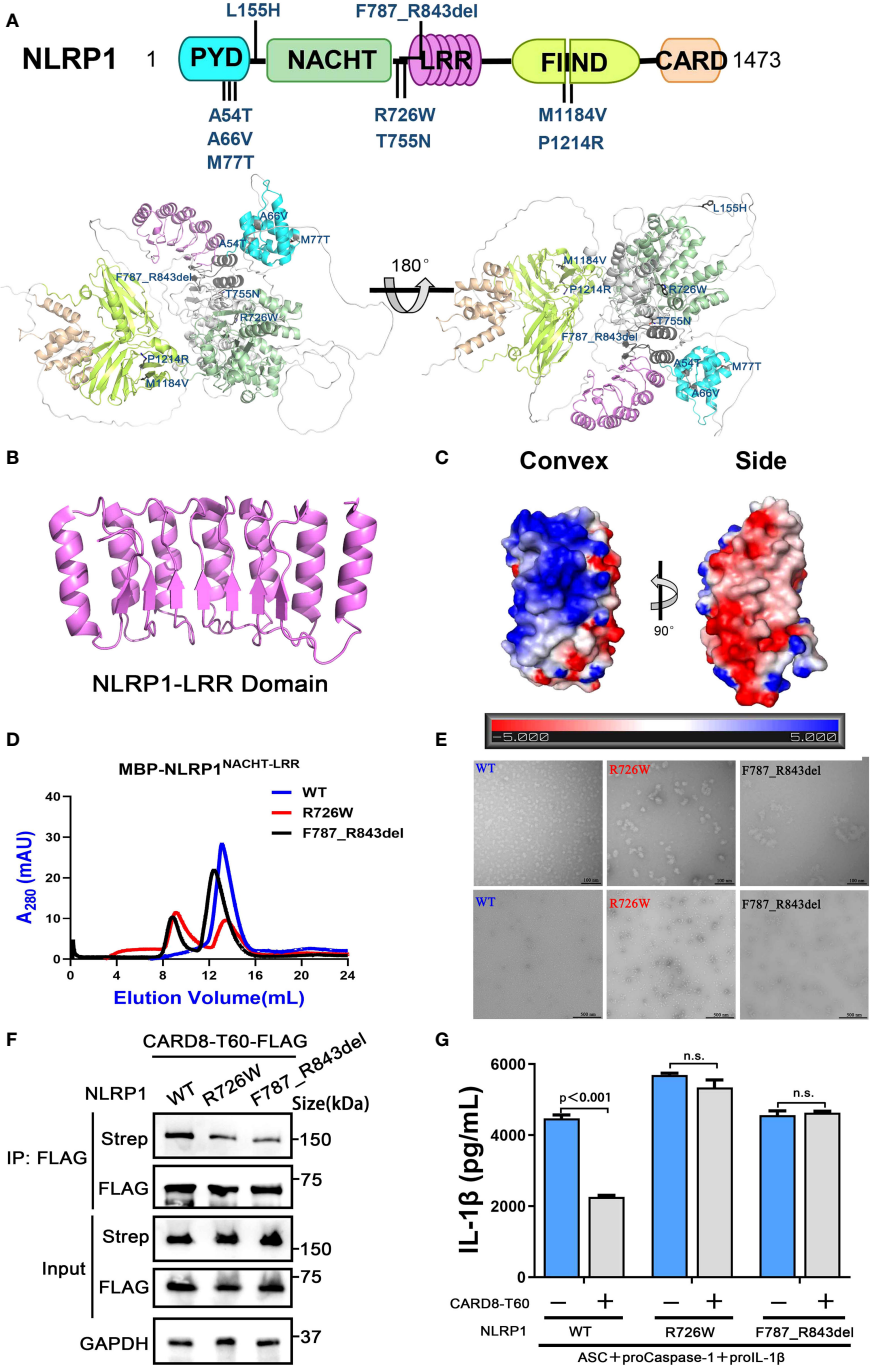
The disease-causing mutations cause decreased interaction between NLRP1 and CARD8. **(A)** The disease-causing mutations in autoimmune-inflammatory diseases are indicated on the predicted structure of full-length NLRP1. **(B)** Monomer crystal structure of LRR domain of NLRP1. **(C)** The electrostatic charge surfaces analysis of NLRP1^LRR^. The electrostatic potential of the convex and side of NLRP1^LRR^ was calculated by APBS. **(D)** Gel filtration characterization of recombinant MBP-NLRP1^NACHT-LRR^, R726W mutant and F787_R843del mutant proteins. **(E)** Representative negative-stain electron microscopy snapshots of recombinant MBP-NLRP1^NACHT-LRR^, R726W mutant and F787_R843del mutant proteins. **(F)** HEK 293T cells were transiently transfected with constructs for NLRP1-Strep, NLRP1-R726W-Strep mutant, NLRP1-F787_R843del-Strep mutant and CARD8-T60-FLAG. After 28 hours, the protein expression was detected by immunoblotting, and co-immunoprecipitation were conducted with anti-FLAG antibody to analyze the interaction. **(G)** HEK 293T cells were transiently transfected with constructs encoding the proIL-1β, proCaspase-1, ASC, CARD8-T60, NLRP1 and NLRP1 mutants. After 48 hours, ELISA was conducted with cell cultural supernatants. Data are shown as mean ± SEM. n.s., not significant.

With the development of whole-exome sequencing technology and further studies of NLRP1 related diseases, an increasing number of mutations of NLRP1 was reported in patients with different auto-inflammatory diseases (29, 36). By summarizing known disease-causing mutations in NLRP1 and mapping NLRP1 mutations onto the predicted full-length structure of NLRP1 (6, 7), we found that these mutations are mainly located in three distinct regions (**Figure 6A**). The auto-inhibitory PYD domain of NLRP1 is the first region that includes A54T, A66V and M77T mutations resulting in rare monogenic inflammatory skin diseases (**Figure S6A**). The second one is localized in the linker region between NACHT domain and LRR domain, including R726W, T755N and F787_R843del mutations (**Figure S6B**). However, the molecular mechanism of how these mutations cause increased NLRP1 activation and autoimmune-inflammatory disease are unknown. M1184V and R1214R mutations located in the third region may affect the auto-processing of FIIND domain to release the C-terminal autoproteolytic fragment of NLRP1 (**Figure S6C**).

Because the NACHT and LRR domains of NLRP1 are critical for the interaction with CARD8-T60 and implicated in several auto-inflammatory diseases, we next sought to study the structure of the NACHT and LRR domains of NLRP1 to elucidate the self-inhibition mechanism of NLRP1. The crystallographic structure of NLRP1^LRR^ revealed a compact structure of crescent shape in 2.45 Å resolution, which is composed of 7 parallel α helices and 7 parallel β strands (**Figure 6B** and **Table S2**). The NLRP1^LRR^ structure is nearly identical to the reported structure (37). Electrostatic surface charge analysis of the NLRP1^LRR^ showed a polar surface pattern, in which the majority of the convex surfaces formed by the α-helices are positively charged, while the surfaces formed by part of the convex and one side are negatively charged (**Figure 6C**). This result is consistent with the observation that NLRP1 could directly bind dsRNA *via* its LRR domain to trigger NLRP1 inflammasome activation (4).

On the other hand, the biochemical properties of R726W and F787_R843del mutations of NLRP1, located in the linker region between NACHT domain and LRR domain, were characterized by size-exclusion chromatography and the negative-stain EM analysis. As expected, the two disease-causing mutations of NLRP1 (R726W and F787_R843del) drastically increase oligomer formation compared with the wild-type NLRP1 NACHT-LRR domain, indicating that these two mutants likely form large oligomers or aggregates (**Figures 6D, E**). More importantly, we note that R726W and F787_R843del mutants, which are implicated in autoimmune and auto-inflammatory diseases, showed a considerably decreased binding with CARD8-T60 (**Figure 6F**). And CARD8-T60 lost the ability of negative regulation NLRP1 inflammasome activation level for R726W and F787_R843del mutants (**Figure 6G**). These findings suggest that mutations at the above regions of NLRP1 may diminish the ability of NLRP1 to associate with the negative regulator CARD8-T60, thus facilitating excessive NLRP1 activation.

## Discussion

The study of the NLRP1 inflammasome, a macromolecular complex platform with NLRP1 as a sensor protein, has witnessed at least two remarkable progresses in recent years. First, it was reported that NLRP1 is activated by two physiological activators, including viral 3C proteases (3) and double-stranded RNA (4). Second, several recent studies have implicated a role for the NLRP1 mutations in multiple autoimmune diseases (36, 38), suggesting the pathogenic mechanisms of many disease-causing mutations. Although human NLRP1, unlike other inflammasome sensors, has a wide tissue expression profile which suggests that it may play essential roles in both host defense and autoinflammatory diseases, the regulatory mechanisms for the NLRP1 inflammasome remain incompletely understood.

The CARD8 gene is capable of generating different isoforms that bear distinct regulatory functions in inflammatory signal transduction. The T60 isoform of CARD8, but not the T48 isoform, inhibits NLRP3 inflammasome activity through binding NLRP3 (20). Consistently, CARD8 was shown to suppress the activation of NLRP3 through the CARD8-NLRP3 interaction. Such regulatory role was lost upon mutations of NLRP3 associated with cryopyrin-associated periodic syndromes (CAPS) (21). Moreover, a recent study proposed that CARD8 disturbs the formation of the NODosome complex *via* the attenuation of the NOD2-mediated oligomerization (19). On the other hand, it is previously reported that DPP8/9 is a binding partner of NLRP1 and maintains NLRP1 in its inactive state to avoid activation of the NLRP1 inflammasome (8). Using cellular overexpression system and biochemical approaches, our results identified CARD8 as a novel inflammasome regulator for human NLRP1 and provided a structural basis for the specificity of signaling pathways in ASC-indispensable, ASC-dispensable and ASC-independent inflammasomes.

**Figure 7:**
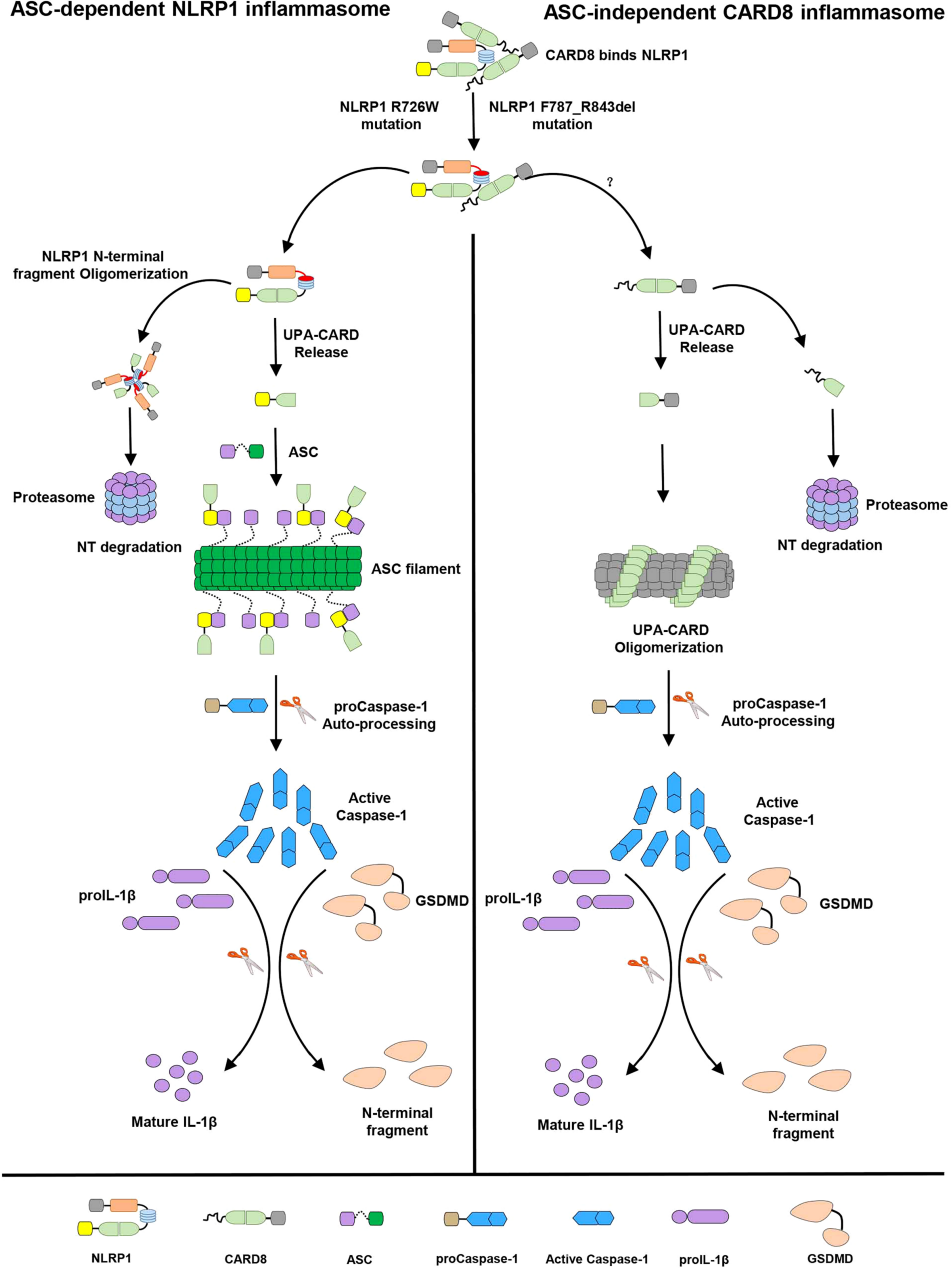
The sequential regulation and activation model of NLRP1 and CARD8 inflammasome. Full-length NLRP1 interacts with CARD8 to maintain the inactive state of NLRP1 and CARD8 in resting cells. The disease-causing mutations would lead to decrease the interaction between NLRP1 and CARD8 and lost the auto-inhibitory mechanism of NLRP1. Then NLRP1 and CARD8 N-terminal degradation by proteasome results in the release of the active C-terminal segment (UPA-CARD domain) from NLRP1 and CARD8. Oligomerized UPA-CARD fragments differentially recruit ASC or CASP1 to assemble the distinct inflammasomes by multiple molecular surfaces of CARDs.

In this study, we identified that the “canonical” T48 isoform of CARD8 interacts with human inflammasome sensor NLRP1, but fails to suppress its activation. Surprisingly, we found the somewhat larger T60 isoform of CARD8 could act as a negative regulator to control the NLRP1 inflammasome activation. Mechanistically, *in vitro* binding assay revealed CARD8-T60 isoform and NLRP1 directly bind to each other. Using a series of truncation mutants of CARD8-T60 isoform and NLRP1, we found that CARD8-T60 variant interacts with NLRP1 through ZU5 subdomain, while NLRP1 interacts with CARD8 through the NACHT, LRR and FIIND domains. In addition, we demonstrated that ASC preference of CARD-containing inflammasomes results from the CARD domain of these receptors, but not the UPA subdomain. Different amino acids in the CARD domain are major contributors of ASC preference and the sixth α-helix of CARD domain does not mediate the hetero-oligomerization in signaling transduction event. Finally, our findings also indicated the patient-derived mutations lead to the NLRP1 oligomerization and disrupt the NLRP1-CARD8 interaction, thereby providing a mechanistic explanation for auto-inflammatory diseases. Although we attempt to offer important details regarding NLRP1-CARD8 interaction and regulation, the study was performed in a transfection system. An appropriate physiologic system would be preferably used to further solidify the current findings. In summary, our cellular, biochemical and structural evidence lead us to propose a model for the signaling pathways involving NLRP1 and CARD8 (**Figure 7**). In resting state, the auto-inhibitory function of the wild-type full-length NLRP1 is maintained by NLRP1 and CARD8 interaction. While these patient-derived NLRP1 mutations could disrupt the self-inhibitory mechanism of NLRP1 by weakening the binding of NLRP1 and CARD8. This triggers constitutive self-oligomerization of NLRP1 and CARD8 and recruits ASC or CASP1 to assemble the distinct inflammasomes which may be mediated by multiple molecular surfaces of CARDs.

The aberrant activation of the NLRP1 inflammasome has been implicated in many auto-inflammatory diseases and the common treatment strategy of these diseases is to block the IL-1β maturation and secretion (39, 40). But the NLRP1 inflammasome also results in cleavage of proIL-18 and pyroptosis, which may be pathogenic and pro-inflammatory. A useful therapy for treating these diseases is to target the upstream signaling molecules to regulate NLRP1 inflammasome activation. Thus, CARD8 and NLRP1 interaction identified by our results might provide a therapeutic target for the treatment of the NLRP1-mediated diseases.

## Materials and methods

### Cloning and reagents

Constructs for entire coding sequence of ASC, proCaspase-1 and IL-1β were prepared as previously described (15). The cDNA sequences of mNLRP1b (UniProt ID: Q2LKW6-1), NLRP1 (UniProt ID: Q9C000-1), CARD8-T48 (UniProt ID: Q9Y2G2-1) and CARD8-T60 (UniProt ID: Q9Y2G2-5) were amplified from mouse or human spleen cDNA libraries and their truncations were constructed by PCR based on the full-length genes. The QuikChange technology was used to generate point mutations of NLRP1 and CARD8. The constructs of UPA-CARD fragment of NLRP1, mNLRP1b and CARD8 were cloned into pACT vector with VP16 activation domain, whereas DNA encoding full-length ASC and truncated ASC were shuttled into pBIND vector with DNA-binding domain and *Renilla reniformis* luciferase sequence. The constructs containing RFP or GFP were used for fluorescence co-localization assay. Other plasmids for expression in HEK 293T cells were inserted into modified pcDNA3.1 vectors using standard cloning techniques. The NLRP1^LRR^ (V790-S990) and NLRP1^NACHT-LRR^ (G230-P994) were engineered into a pET30a-derived vector with a Tobacco Etch Virus (TEV) protease linker between N-terminal fusion tag and target protein *via* homologous recombination. All plasmids were confirmed by Sanger sequencing. The reagents used include Human IL-1β ELISA kit (BD Biosciences, 557953), anti-FLAG antibody (Sigma, F1804), anti-Strep (Biolegend, 688202), anti-GAPDH (ABclonal, AC002), anti-His (Sangon Biotech, D191001), anti-GFP (Abmart, M20004M), FLAG Beads (Sigma, A2220), Strep Beads (IBA, 2-1208-025), Z-VAD-FMK (APExBio, A1902), Val-boroPro (TargetMol, T4042), Disuccinimidyl suberate (DSS) (PIERCE, 21555).

### Cell culture and stable cell lines generation

HEK 293T cells were purchased from Cell Bank of Chinese Academy of Sciences and grown in Dulbecco’s modified Eagle’s medium (DMEM) supplemented with 10% FBS, 100 U/ml penicillin and 100 μg/ml streptomycin. Transfection of HEK 293T with 2 μg of ASC-GFP-pCDH, 2 μg psPAX2 and 2 μg pMD2.G was performed to package the lentivirus. The virus supernatants with 10 μg/ml polybrene were used to infect HEK 293T cells. 36 hours later, the cells were selected for ectopically expressing ASC-GFP HEK 293T cell line using 5 μg/ml puromycin. All cells were cultured at 37 °C in an incubator containing 5% CO_2_.

### Determination of cleaved IL-1β of NLRP1 inflammasome activation assay

HEK 293T cells were plated at 1×10^5^ per well in twenty-four wells plates overnight before transfection. Then each well of cells was co-transfected with NLRP1, ASC, Caspase-1 and IL-1β plasmids to constitute NLRP1 inflammasome using lipo6000™ at a “20:10:2:35” ratio according to the instructions of manufacturer. 24 hours after transfection, the media was changed with fresh medium for another 24 hours. The concentrations of IL-1β in cell lysates were measured by immunoblotting. Determination of secreted IL-1β in cell culture supernatants was performed with ELISA kits (BD Biosciences) according to standard manufacturer’s instructions without modification.

### SDS-PAGE, immunoblotting and immunoprecipitation

Cells were treated with ice-cold lysis buffer (20 mM Tris-HCl pH 7.5, 100 mM KCl, 5 mM MgCl_2_, 0.3% NP-40 and protease inhibitor cocktail) for 30 mins and centrifuged at 12000 rpm for 15 mins. The soluble fraction was incubated with Anti-FLAG M2 agarose beads or Strep-Tactin superflow affinity gel overnight at 4 °C. After the gels were washed three times with lysis buffer, the bound proteins were eluted by boiling, separated by 15% SDS-PAGE, and visualized by Coomassie blue staining or immunoblotting. For immunoblotting, the PVDF membranes were used to transfer proteins, blocked by 5% non-fat milk, and incubated with respective antibodies at 4 °C. The protein bands were developed with ECL reagents and visualized by Bio-Rad Gel Doc System.

### Fluorescence co-localization and ASC-GFP speck quantification assay

A density of 5×10^5^ cells was seeded on glass-bottom dishes, followed by fixedness with 4% paraformaldehyde 26 hours post transfection as described. Live-cells were incubated with 1 μg/ml DAPI for the sake of nucleus staining and visualized using a fluorescence microscope (OLYMPUS, TH4-200). For ASC-GFP speck quantification assay, total cell area was counted using blue fluorescence channel images. Quantification of ASC Speck was performed by the “Analyze particles” function following threshold adjustment based on GFP-positive signal in ImageJ/FlJl software.

### Mammalian two-hybrid assay

Based on Checkmate mammalian two-hybrid system (Promega, Madison, WI, USA), the indicated genes were engineered into pACT and pBIND vectors, respectively. These pACT, pBIND and pG5*luc* plasmids at 1:1:1 molar ration were co-transfected into HEK 293T cells following the manufacturer’s manual. The relative luciferase activity was determined by Dual Luciferase Reporter Gene Assay Kit (Beyotime) after 24 hours post transfection. The empty plasmids were also transfected into cells as a negative control and three independent experiments were performed for data acquisition.

### Recombinant protein preparation

Recombinant NLRP1^LRR^ tagged with a removable GB-1 was expressed following Studier and colleagues’ protocol (41). The BL-21-CodonPlus RIPL strain (Strategen) was transformed with expression plasmids encoding NLRP1^LRR^ protein. Bacteria were grown in LB medium and induced with 0.3 mM isopropyl 1-thio-β-D-galactopyranoside (IPTG) when OD_600_ reached 0.6-0.8 at 16 °C. The bacterial samples were harvested by centrifugation at 15000 rpm for 25 min and lysed by sonication, followed by Ni-NTA chromatography. The recombinant GB-1- NLRP1^LRR^ protein was added with TEV protease to remove the GB-1 tag and separated by the second step of Ni-NTA purification. The non-tagged NLRP1^LRR^ protein was further purified from eluant to homogeneity by size exclusion chromatography with a soluble buffer containing 100 mM NaCl, 5% glycerol, and 20 mM HEPES pH 7.4.

Expression and purification of NLRP1^NACHT-LRR^ and its mutated proteins were similar to the above. Following sonication and centrifugation in lysis buffer (50 mM Tris-HCl at pH 7.5, 10% glycerol, 500 mM NaCl, 20 mM imidazole and 0.01% NP40), the clarified supernatant containing the NLRP1^NACHT-LRR^ protein with maltose-binding protein (MBP) tag was purified on Hisprep IMAC column (GE Healthcare) and eluted with elution buffer contained 50 mM Tris-HCl at pH 7.5, 10% glycerol, 500 mM NaCl, 300 mM imidazole and 0.01% NP40. To further purify the final MBP-NLRP1^NACHT-LRR^ protein, size exclusion chromatography was performed with an XK26/60 Superdex 200 size exclusion column in 50 mM Tris-HCl at pH 7.5, 10% glycerol, 300 mM NaCl and 0.01% NP40 buffer.

### Negative-stain EM

In brief, the carbon coated 400-mesh Cu EM specimen grids (Solarus, Gatan, Model 950) were glow discharged before 50-100 μg/ml of protein samples were left on the grids for 90 s. After the samples were blotted, stain containing 2% Uranyl Acetate was employed to incubate with the grids for 60s. Following air dry of the grids, negative stained images were taken by 120 keV electron microscopy.

### Size-exclusion chromatography assay

To assess the oligomeric state of NLRP1^NACHT-LRR^ and its mutated proteins, the protein samples were subjected to analytical gel filtration using an XK26/60 Superdex 200 size exclusion column equilibrated with a buffer containing 50 mM Tris-HCl at pH 7.5, 10% glycerol, 300 mM NaCl and 0.01% NP40.

### Crystallography

The ~50 mg/ml NLRP1^LRR^ protein was mixed at a 1:1 ratio with reservoir solution for crystallization conditions screening. Diffraction quality crystals were obtained by hanging-drop vapor-diffusion method with reservoir condition of 1.6 M Sodium Potassium Phosphate, 100 mM HEPES, pH 6.4. Typical diamond shaped crystals of 0.3-0.5 µm in each dimension grow in 2 weeks at room temperature. A final concentration of 20% glycerol was added into the crystallization condition as a cryoprotectant solution before flash-cooled the crystals in liquid nitrogen for data collection. X-ray diffraction data were collected at beamline X12C, X29A and X25 at the National Light Source, the Brookhaven National Laboratory and beamline SER-CAT 22ID and GM-CA 23ID at the Advanced Photon Source (APS), the Argonne National Laboratory. Data were processed with *HKL2000* (42) and XDS (43). In order to solve the phase problem, a homology structure model was built with the Swiss-Model server (44) using the crystal structure of ribonuclease inhibitor (PDB: 1DFJ) (45) as template. Molecular replacement was calculated in *Phaser* (46) in the CCP4i GUI (47) by using 4 central repeats of the LRR homology model as an initial search model. The structure model was completed by manual building in *Coot* (48). Structure refinement was performed by REFMAC5 (49), CNS (50) and PHENIX (51). Structural figures were prepared by *Pymol*.

### Statistical analysis

GraphPad Prism 8.0 software was used for statistical analysis and error bars were plotted as mean ± SEM. Statistically significant of three independent biological experiments was determined by student’s unpaired *t*-test, and *P*-value less than 0.05 was denoted significant.

### Accession number

The accession number for coordinates and structural factors of NLRP1^LRR^ in this study is 5Y3S. The data of this manuscript can be obtained from the corresponding author upon request.

## Data availability statement

The original contributions presented in the study are included in the article/**Supplementary Material**. Further inquiries can be directed to the corresponding authors.

## Author contributions

ZHX designed, carried out almost all experiments and wrote the first draft of the paper. SSD, YLH, YRY, DZ, YYW and PYZ collected, analyzed and interpreted the data. TSX, LQS, WHZ, XYY, MZYL, HYL, and WDZ contributed to the helpful discussion and comment on experiment results. YZ and TCJ provided study design, supervision and data analysis, and approved the final version of the manuscript.

## Funding

This study is supported by Strategic Priority Research Program of the Chinese Academy of Sciences (Grant No. XDB29030104), the National Natural Science Foundation of China (Grant No. 82272301, 81872110 and 82172773), the Fundamental Research Funds for the Central Universities (WK9100000001).

## Conflict of interest

The authors declare that the research was conducted in the absence of any commercial or financial relationships that could be construed as a potential conflict of interest.

## Publisher’s note

All claims expressed in this article are solely those of the authors and do not necessarily represent those of their affiliated organizations, or those of the publisher, the editors and the reviewers. Any product that may be evaluated in this article, or claim that may be made by its manufacturer, is not guaranteed or endorsed by the publisher.

## Supplementary material

Supplementary Figure 1 Protein-protein interaction network of the inflammasome scaffold molecules. Different line colors represent different types of protein associations. Experimental (red), Curated databases (light blue), Protein homology (purple).

Supplementary Figure 2 Generation of the HEK 293T cell line stably expressing ASC-GFP. Efficiency of knock-in was evaluated using western blot.

Supplementary Figure 3 Physical interaction between NLRP1 and CARD8. **(A)** Transient transfection RFP-CARD8 and mNLRP1b-GFP in HEK 293T cells. Green and red fluorescent were captured by fluorescence microscopy. Co-localization regions are labeled with white arrow. **(B)** Colocalization coefficient of CARD8 with NLRP1 or mNLRP1b in HEK 293T cells (n=15 cells from three biological replicates). **(C)** Schematic drawing of the pull-down strategy by anti-FLAG and anti-Strep antibody for detection of NLRP1 and CARD8 direct interaction.

Supplementary Figure 4 Distinct assembly mechanism for ASC dependence in NLRP1 and CARD8 inflammasome. **(A, B)** ELISA and immunoblotting analysis were conducted from ASC-deficient NLRP1, mNLRP1b and CARD8 inflammasome. Data are shown as mean ± SEM and representative of three biological replicates. **(C, D)** HEK 293T cells stably expressing ASC-GFP were transiently transfected with the indicated expression plasmids and treated with VbP (5 μM) for 6 hours. The cells were fixed with 4% formaldehyde and evaluated for ASC speck formation by fluorescence microscopy in three differential regions. Data of mean specks per cell area are shown as mean ± SEM in c and representative images are shown in d.

Supplementary Figure 5 NLRP1 CARD domain mutations analysis using NLRP1 inflammasome reconstitution assay in HEK 293T cells. **(A)** Immunoblotting analysis were conducted from wild-type, A1-1385, A1-1409/1410 and A1-1415/1417 mutations of NLRP1^UPA-CARD^ in reconstituted NLRP1 inflammasome system. **(B)** Immunoblotting analysis were conducted from A1-1423/1425, A1-1434, A1-1437/1439 and B1-F4M mutations of NLRP1^UPA-CARD^ in reconstituted NLRP1 inflammasome system. **(C)** Immunoblotting analysis were conducted from B1-F4M2, B1-F5M, B1-F6M and B1-F6M-1397-1399 mutations of NLRP1^UPA-CARD^ in reconstituted NLRP1 inflammasome system. **(D)** Immunoblotting analysis were conducted from B1-F6M-1422-1425, B1-F6M-1443-1444, C1-F6M-S2M and C1-F6M-S3M mutations of NLRP1^UPA-CARD^ in reconstituted NLRP1 inflammasome system.

Supplementary Figure 6 Sequence alignment analysis of NLRP1 from indicated species. **(A)** Sequence alignment analysis of PYD domain of NLRP1 from indicated species. Disease-causing mutations are highlighted in red. **(B)** Sequence alignment analysis of interdomain linker between NLRP1^NACHT^ domain and NLRP1^LRR^ domain from indicated species. Disease-causing mutations are highlighted in red or marked with gray shade. **(C)** Sequence alignment analysis of interdomain sequences between NLRP1^ZU5^ subdomain and NLRP1^UPA^ subdomain from indicated species. Disease-causing mutations are highlighted in red and proteolytic site is marked with green triangle arrow.

## Abbreviations

proIL-1β, pro-interleukin-1β; proIL-18, pro-interleukin-18; GSDMD, gasdermin D; PAMPs, pathogen-associated molecular patterns; VbP, Val-boroPro; AIADK, autoinflammation with arthritis and dyskeratosis; FKLC, familial keratosis lichenoides chronica; FIIND, function-to-find domain; CARD, caspase activation and recruitment domain; ZU5, found in ZO-1 and UNC5; UPA, conserved in UNC5, PIDD, and ankyrins; DD, death domain; CAPS, cryopyrin-associated periodic syndromes; TEV, Tobacco Etch Virus; MBP, maltose-binding protein.

## References

[1] Lamkanfi M Dixit VM. Mechanisms and functions of inflammasomes. Cell (2014) 157:1013–22. doi: 10.1016/j.cell.2014.04.007 24855941

[2] Martinon F Burns K Tschopp J. The inflammasome: a molecular platform triggering activation of inflammatory caspases and processing of proIL-beta. Mol Cell (2002) 10:417–26. doi: 10.1016/s1097-2765(02)00599-3 12191486

[3] Robinson KS Teo DET Tan KS Toh GA Ong HH Lim CK. Enteroviral 3C protease activates the human NLRP1 inflammasome in airway epithelia. Science (2020) 370:eaay2002. doi: 10.1126/science.aay2002 33093214

[4] Bauernfried S Scherr MJ Pichlmair A Duderstadt KE Hornung V. Human NLRP1 is a sensor for double-stranded RNA. Science (2021) 371:eabd0811. doi: 10.1126/science.abd0811 33243852

[5] Sharif H Hollingsworth LR Griswold AR Hsiao JC Wang Q Bachovchin DA. Dipeptidyl peptidase 9 sets a threshold for CARD8 inflammasome formation by sequestering its active c-terminal fragment. Immunity (2021) 54:1392–1404 e10. doi: 10.1016/j.immuni.2021.04.024 34019797 PMC8423358

[6] Huang M Zhang X Toh GA Gong Q Wang J Han Z. Structural and biochemical mechanisms of NLRP1 inhibition by DPP9. Nature (2021) 592:773–7. doi: 10.1038/s41586-021-03320-w PMC8081665 33731929

[7] Hollingsworth LR Sharif H Griswold AR Fontana P Mintseris J Dagbay KB. DPP9 sequesters the c terminus of NLRP1 to repress inflammasome activation. Nature (2021) 592:778–83. doi: 10.1038/s41586-021-03350-4 PMC8299537 33731932

[8] Zhong FL Robinson K Teo DET Tan KY Lim C Harapas CR. Human DPP9 represses NLRP1 inflammasome and protects against autoinflammatory diseases *via* both peptidase activity and FIIND domain binding. J Biol Chem (2018) 293:18864–78. doi: 10.1074/jbc.RA118.004350 PMC6295727 30291141

[9] Johnson DC Taabazuing CY Okondo MC Chui AJ Rao SD Brown FC. DPP8/DPP9 inhibitor-induced pyroptosis for treatment of acute myeloid leukemia. Nat Med (2018) 24:1151–6. doi: 10.1038/s41591-018-0082-y PMC6082709 29967349

[10] Sandstrom A Mitchell PS Goers L Mu EW Lesser CF Vance RE. Functional degradation: A mechanism of NLRP1 inflammasome activation by diverse pathogen enzymes. Science (2019) 364:eaau1330. doi: 10.1126/science.aau1330 30872533 PMC6532986

[11] Chui AJ Okondo MC Rao SD Gai K Griswold AR Johnson DC. N-terminal degradation activates the NLRP1B inflammasome. Science (2019) 364:82–5. doi: 10.1126/science.aau1208 PMC6610862 30872531

[12] Grandemange S Sanchez E Louis-Plence P Tran Mau-Them F Bessis D Coubes C. A new autoinflammatory and autoimmune syndrome associated with NLRP1 mutations: NAIAD (NLRP1-associated autoinflammation with arthritis and dyskeratosis). Ann Rheum Dis (2017) 76:1191–8. doi: 10.1136/annrheumdis-2016-210021 27965258

[13] Zhong FL Mamai O Sborgi L Boussofara L Hopkins R Robinson K. Germline NLRP1 mutations cause skin inflammatory and cancer susceptibility syndromes *via* inflammasome activation. Cell (2016) 167:187–202 e17. doi: 10.1016/j.cell.2016.09.001 27662089

[14] Ball DP Taabazuing CY Griswold AR Orth EL Rao SD Kotliar IB. Caspase-1 interdomain linker cleavage is required for pyroptosis. Life Sci Alliance (2020) 3:e202000664. doi: 10.26508/lsa.202000664 32051255 PMC7025033

[15] Xu Z Zhou Y Liu M Ma H Sun L Zahid A. Homotypic CARD-CARD interaction is critical for the activation of NLRP1 inflammasome. Cell Death Dis (2021) 12:57. doi: 10.1038/s41419-020-03342-8 33431827 PMC7801473

[16] Poyet JL Srinivasula SM Tnani M Razmara M Fernandes-Alnemri T Alnemri ES. Identification of ipaf, a human caspase-1-activating protein related to apaf-1. J Biol Chem (2001) 276:28309–13. doi: 10.1074/jbc.C100250200 11390368

[17] Zhang L Chen S Ruan J Wu J Tong AB Yin Q. Cryo-EM structure of the activated NAIP2-NLRC4 inflammasome reveals nucleated polymerization. Science (2015) 350:404–9. doi: 10.1126/science.aac5789 PMC4640189 26449474

[18] Xu H Shi J Gao H Liu Y Yang Z Shao F. The n-end rule ubiquitin ligase UBR2 mediates NLRP1B inflammasome activation by anthrax lethal toxin. EMBO J (2019) 38:e101996. doi: 10.15252/embj.2019101996 31268597 PMC6600268

[19] Von Kampen O Lipinski S Till A Martin SJ Nietfeld W Lehrach H. Caspase recruitment domain-containing protein 8 (CARD8) negatively regulates NOD2-mediated signaling. J Biol Chem (2010) 285:19921–6. doi: 10.1074/jbc.M110.127480 PMC2888403 20385562

[20] Mao L Kitani A Similuk M Oler AJ Albenberg L Kelsen J. Loss-of-function CARD8 mutation causes NLRP3 inflammasome activation and crohn's disease. J Clin Invest (2018) 128:1793–806. doi: 10.1172/JCI98642 PMC5919822 29408806

[21] Ito S Hara Y Kubota T. CARD8 is a negative regulator for NLRP3 inflammasome, but mutant NLRP3 in cryopyrin-associated periodic syndromes escapes the restriction. Arthritis Res Ther (2014) 16:R52. doi: 10.1186/ar4483 24517500 PMC4060228

[22] Wang Q Gao H Clark KM Mugisha CS Davis K Tang JP. CARD8 is an inflammasome sensor for HIV-1 protease activity. Science (2021) 371:eabe1707. doi: 10.1126/science.abe1707 33542150 PMC8029496

[23] Chui AJ Griswold AR Taabazuing CY Orth EL Gai K Rao SD. Activation of the CARD8 inflammasome requires a disordered region. Cell Rep (2020) 33:108264. doi: 10.1016/j.celrep.2020.108264 33053349 PMC7594595

[24] Szklarczyk D Gable AL Lyon D Junge A Wyder S Huerta-Cepas J. STRING v11: protein-protein association networks with increased coverage, supporting functional discovery in genome-wide experimental datasets. Nucleic Acids Res (2019) 47:D607–13. doi: 10.1093/nar/gky1131 PMC6323986 30476243

[25] Fagerberg L Hallstrom BM Oksvold P Kampf C Djureinovic D Odeberg J. Analysis of the human tissue-specific expression by genome-wide integration of transcriptomics and antibody-based proteomics. Mol Cell Proteomics (2014) 13:397–406. doi: 10.1074/mcp.M113.035600 24309898 PMC3916642

[26] Bagnall RD Roberts RG Mirza MM Torigoe T Prescott NJ Mathew CG. Novel isoforms of the CARD8 (TUCAN) gene evade a nonsense mutation. Eur J Hum Genet (2008) 16:619–25. doi: 10.1038/sj.ejhg.5201996 18212821

[27] Van Opdenbosch N Gurung P Vande Walle L Fossoul A Kanneganti TD Lamkanfi M. Activation of the NLRP1b inflammasome independently of ASC-mediated caspase-1 autoproteolysis and speck formation. Nat Commun (2014) 5:3209. doi: 10.1038/ncomms4209 24492532 PMC3926011

[28] Gong Q Robinson K Xu C Huynh PT Chong KHC Tan EYJ. Structural basis for distinct inflammasome complex assembly by human NLRP1 and CARD8. Nat Commun (2021) 12:188. doi: 10.1038/s41467-020-20319-5 33420028 PMC7794362

[29] Taabazuing CY Griswold AR Bachovchin DA. The NLRP1 and CARD8 inflammasomes. Immunol Rev (2020) 297:13–25. doi: 10.1111/imr.12884 32558991 PMC7483925

[30] Jin T Curry J Smith P Jiang J Xiao TS. Structure of the NLRP1 caspase recruitment domain suggests potential mechanisms for its association with procaspase-1. Proteins (2013) 81:1266–70. doi: 10.1002/prot.24287 PMC3860829 23508996

[31] Jin T Huang M Smith P Jiang J Xiao TS. The structure of the CARD8 caspase-recruitment domain suggests its association with the FIIND domain and procaspases through adjacent surfaces. Acta Crystallogr Sect F Struct Biol Cryst Commun (2013) 69:482–7. doi: 10.1107/S1744309113010075 PMC3660883 23695559

[32] Jumper J Evans R Pritzel A Green T Figurnov M Ronneberger O. Highly accurate protein structure prediction with AlphaFold. Nature (2021) 596:583–9. doi: 10.1038/s41586-021-03819-2 PMC8371605 34265844

[33] Varadi M Anyango S Deshpande M Nair S Natassia C Yordanova G. AlphaFold protein structure database: massively expanding the structural coverage of protein-sequence space with high-accuracy models. Nucleic Acids Res (2022) 50:D439–44. doi: 10.1093/nar/gkab1061 PMC8728224 34791371

[34] Lin SC Lo YC Wu H. Helical assembly in the MyD88-IRAK4-IRAK2 complex in TLR/IL-1R signalling. Nature (2010) 465:885–90. doi: 10.1038/nature09121 PMC2888693 20485341

[35] Robert Hollingsworth L David L Li Y Griswold AR Ruan J Sharif H. Mechanism of filament formation in UPA-promoted CARD8 and NLRP1 inflammasomes. Nat Commun (2021) 12:189. doi: 10.1038/s41467-020-20320-y 33420033 PMC7794386

[36] Fenini G Karakaya T Hennig P Di Filippo M Beer HD. The NLRP1 inflammasome in human skin and beyond. Int J Mol Sci (2020) 21. doi: 10.3390/ijms21134788 PMC7370280 32640751

[37] Reubold TF Hahne G Wohlgemuth S Eschenburg S. Crystal structure of the leucine-rich repeat domain of the NOD-like receptor NLRP1: implications for binding of muramyl dipeptide. FEBS Lett (2014) 588:3327–32. doi: 10.1016/j.febslet.2014.07.017 25064844

[38] Tupik JD Nagai-Singer MA Allen IC. To protect or adversely affect? the dichotomous role of the NLRP1 inflammasome in human disease. Mol Aspects Med (2020) 76:100858. doi: 10.1016/j.mam.2020.100858 32359693

[39] So A De Smedt T Revaz S Tschopp J. A pilot study of IL-1 inhibition by anakinra in acute gout. Arthritis Res Ther (2007) 9:R28. doi: 10.1186/ar2143 17352828 PMC1906806

[40] Mertens M Singh JA. Anakinra for rheumatoid arthritis: a systematic review. J Rheumatol (2009) 36:1118–25. doi: 10.3899/jrheum.090074 19447938

[41] Studier FW. Protein production by auto-induction in high density shaking cultures. Protein Expr Purif (2005) 41:207–34. doi: 10.1016/j.pep.2005.01.016 15915565

[42] Otwinowski Z Minor W. Processing of X-ray diffraction data collected in oscillation mode. Methods Enzymol (1997) 276:307–26. doi: 10.1016/S0076-6879(97)76066-X 27754618

[43] Kabsch W. Xds. Acta Crystallogr D Biol Crystallogr (2010) 66:125–32. doi: 10.1107/S0907444909047337 PMC2815665 20124692

[44] Arnold K Bordoli L Kopp J Schwede T. The SWISS-MODEL workspace: a web-based environment for protein structure homology modelling. Bioinformatics (2006) 22:195–201. doi: 10.1093/bioinformatics/bti770 16301204

[45] Kobe B Deisenhofer J. A structural basis of the interactions between leucine-rich repeats and protein ligands. Nature (1995) 374:183–6. doi: 10.1038/374183a0 7877692

[46] Mccoy AJ Grosse-Kunstleve RW Adams PD Winn MD Storoni LC Read RJ. Phaser crystallographic software. J Appl Crystallogr (2007) 40:658–74. doi: 10.1107/S0021889807021206 PMC2483472 19461840

[47] Potterton E Briggs P Turkenburg M Dodson E. A graphical user interface to the CCP4 program suite. Acta Crystallogr D Biol Crystallogr (2003) 59:1131–7. doi: 10.1107/s0907444903008126 12832755

[48] Emsley P Lohkamp B Scott WG Cowtan K. Features and development of coot. Acta Crystallogr D Biol Crystallogr (2010) 66:486–501. doi: 10.1107/S0907444910007493 20383002 PMC2852313

[49] Murshudov GN Vagin AA Dodson EJ. Refinement of macromolecular structures by the maximum-likelihood method. Acta Crystallogr D Biol Crystallogr (1997) 53:240–55. doi: 10.1107/S0907444996012255 15299926

[50] Brunger AT Adams PD Clore GM Delano WL Gros P Grosse-Kunstleve RW. Crystallography & NMR system: A new software suite for macromolecular structure determination. Acta Crystallogr D Biol Crystallogr (1998) 54:905–21. doi: 10.1107/s0907444998003254 9757107

[51] Adams PD Afonine PV Bunkoczi G Chen VB Davis IW Echols N. PHENIX: a comprehensive Python-based system for macromolecular structure solution. Acta Crystallogr D Biol Crystallogr (2010) 66:213–21. doi: 10.1107/S0907444909052925 PMC2815670 20124702

