# Supplementary figures and images for: The CARD8 T60 variant associates with NLRP1 and negatively regulates its activation
Source: Front Immunol. 2022 Nov 8;13:1047922. doi: 10.3389/fimmu.2022.1047922 (PMC9679424; doi:10.3389/fimmu.2022.1047922)

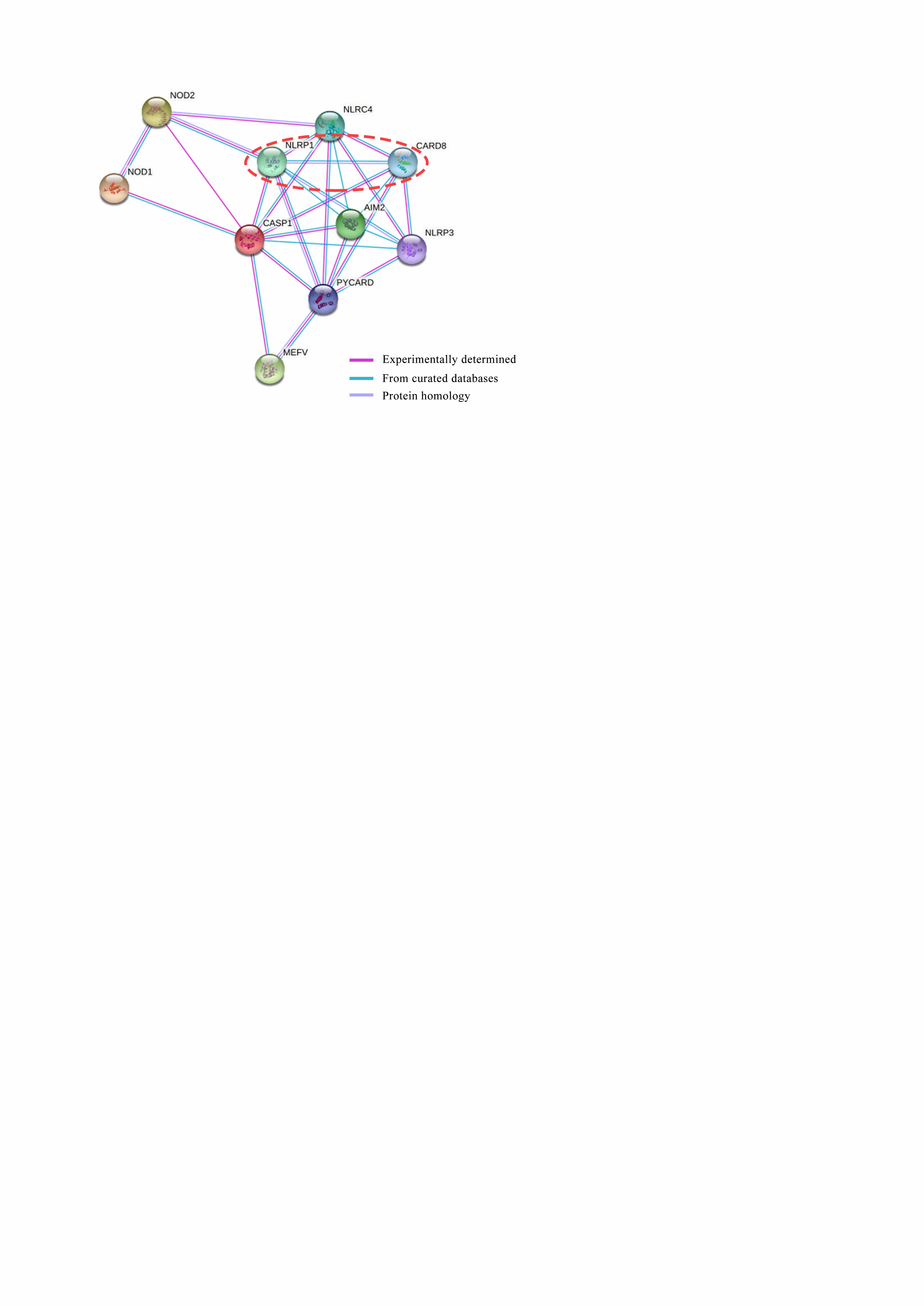

Supplement: Supplementary Figure 1 — Protein-protein interaction network of the inflammasome scaffold molecules. Different line colors represent different types of protein associations. Experimental (red), Curated databases (light blue), Protein homology (purple). [file Image_1.jpeg]

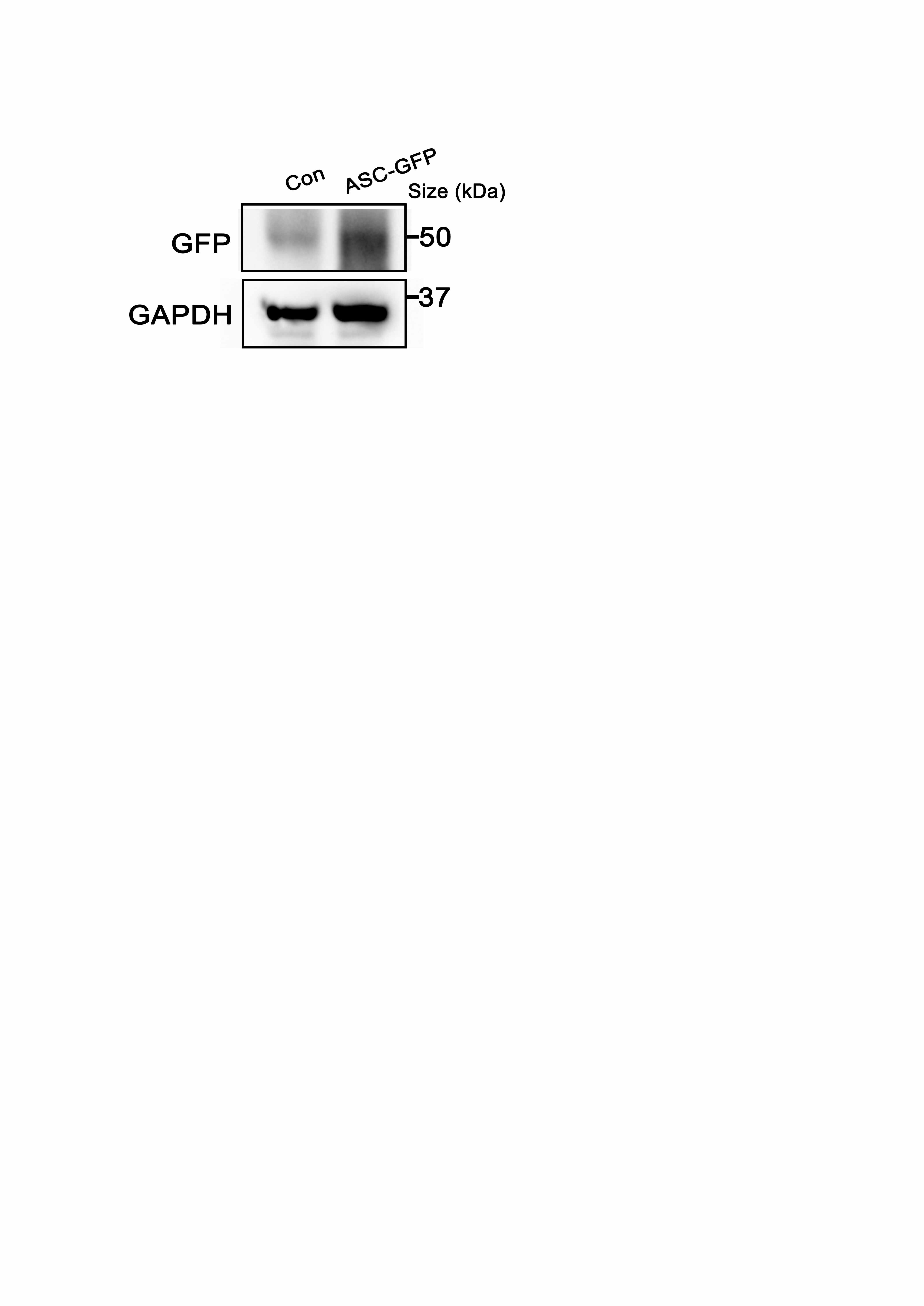

Supplement: Supplementary Figure 2 — Generation of the HEK 293T cell line stably expressing ASC-GFP. Efficiency of knock-in was evaluated using western blot. [file Image_2.jpeg]

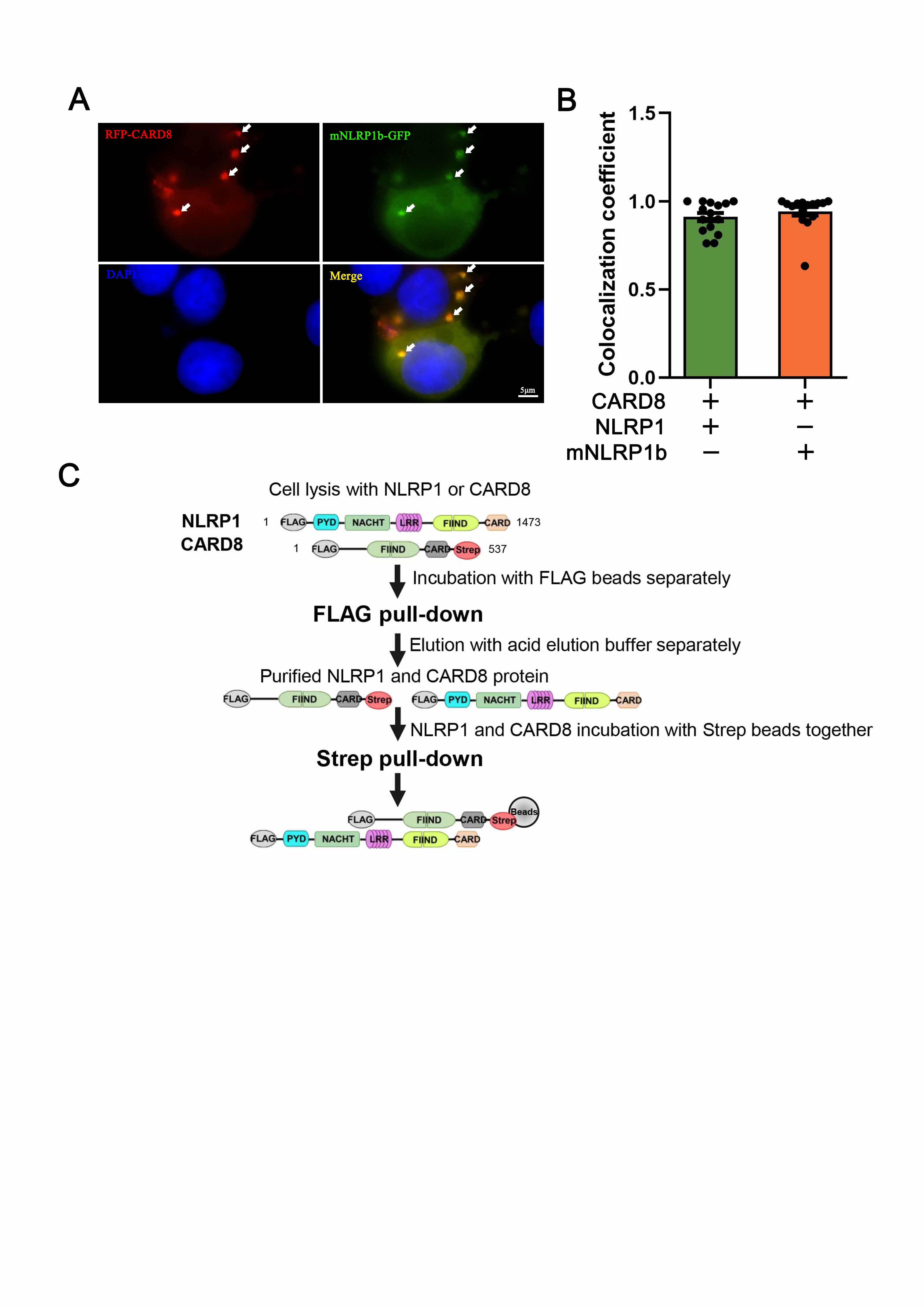

Supplement: Supplementary Figure 3 — Physical interaction between NLRP1 and CARD8. (A) Transient transfection RFP-CARD8 and mNLRP1b-GFP in HEK 293T cells. Green and red fluorescent were captured by fluorescence microscopy. Co-localization regions are labeled with white arrow. (B) Colocalization coefficient of CARD8 with NLRP1 or mNLRP1b in HEK 293T cells (n=15 cells from three biological replicates). (C) Schematic drawing of the pull-down strategy by anti-FLAG and anti-Strep antibody for detection of NLRP1 and CARD8 direct interaction. [file Image_3.jpeg]

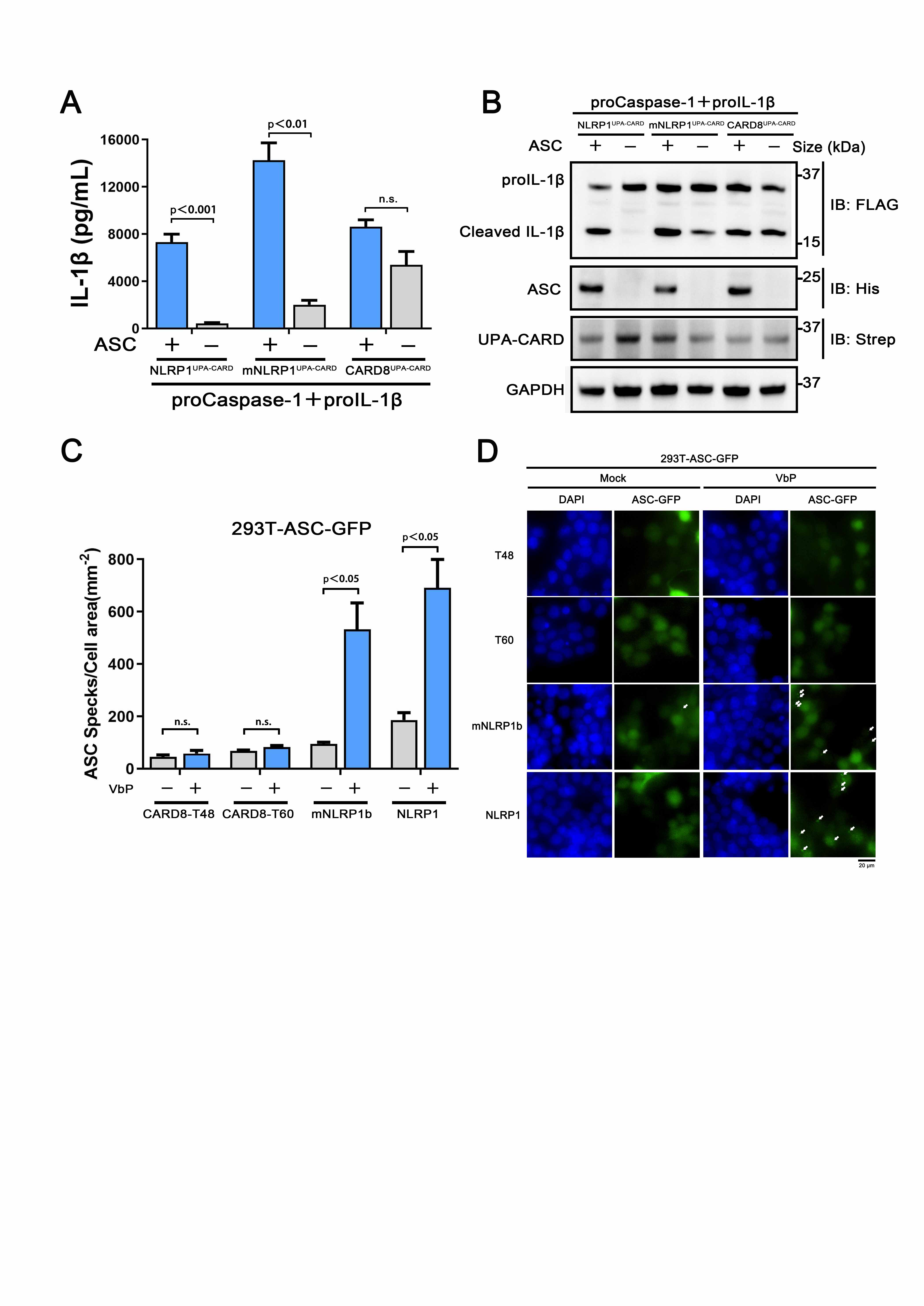

Supplement: Supplementary Figure 4 — Distinct assembly mechanism for ASC dependence in NLRP1 and CARD8 inflammasome. (A, B) ELISA and immunoblotting analysis were conducted from ASC-deficient NLRP1, mNLRP1b and CARD8 inflammasome. Data are shown as mean ± SEM and representative of three biological replicates. (C, D) HEK 293T cells stably expressing ASC-GFP were transiently transfected with the indicated expression plasmids and treated with VbP (5 μM) for 6 hours. The cells were fixed with 4% formaldehyde and evaluated for ASC speck formation by fluorescence microscopy in three differential regions. Data of mean specks per cell area are shown as mean ± SEM in c and representative images are shown in d. [file Image_4.jpeg]

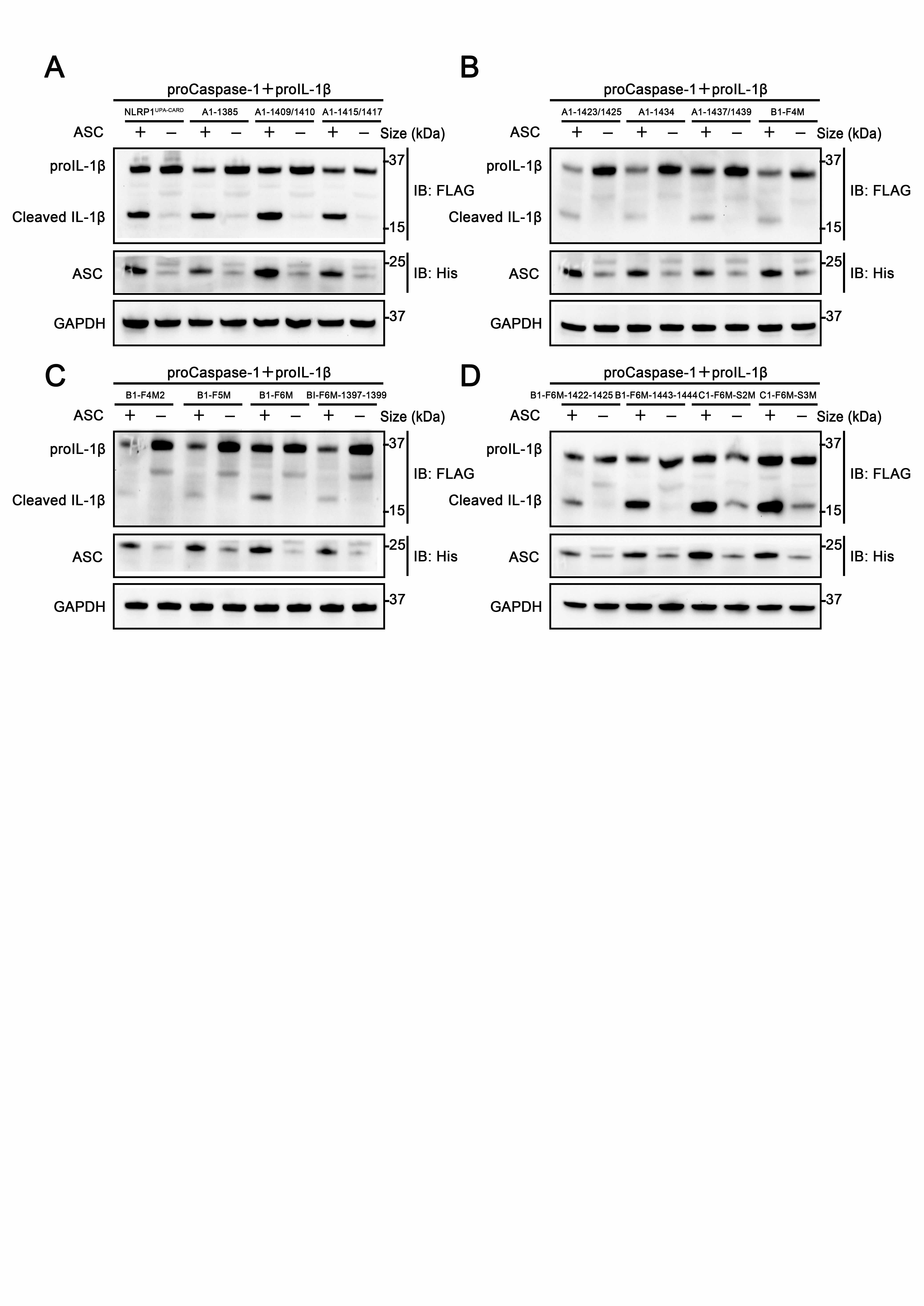

Supplement: Supplementary Figure 5 — NLRP1 CARD domain mutations analysis using NLRP1 inflammasome reconstitution assay in HEK 293T cells. (A) Immunoblotting analysis were conducted from wild-type, A1-1385, A1-1409/1410 and A1-1415/1417 mutations of NLRP1UPA-CARD in reconstituted NLRP1 inflammasome system. (B) Immunoblotting analysis were conducted from A1-1423/1425, A1-1434, A1-1437/1439 and B1-F4M mutations of NLRP1UPA-CARD in reconstituted NLRP1 inflammasome system. (C) Immunoblotting analysis were conducted from B1-F4M2, B1-F5M, B1-F6M and B1-F6M-1397-1399 mutations of NLRP1UPA-CARD in reconstituted NLRP1 inflammasome system. (D) Immunoblotting analysis were conducted from B1-F6M-1422-1425, B1-F6M-1443-1444, C1-F6M-S2M and C1-F6M-S3M mutations of NLRP1UPA-CARD in reconstituted NLRP1 inflammasome system. [file Image_5.jpeg]

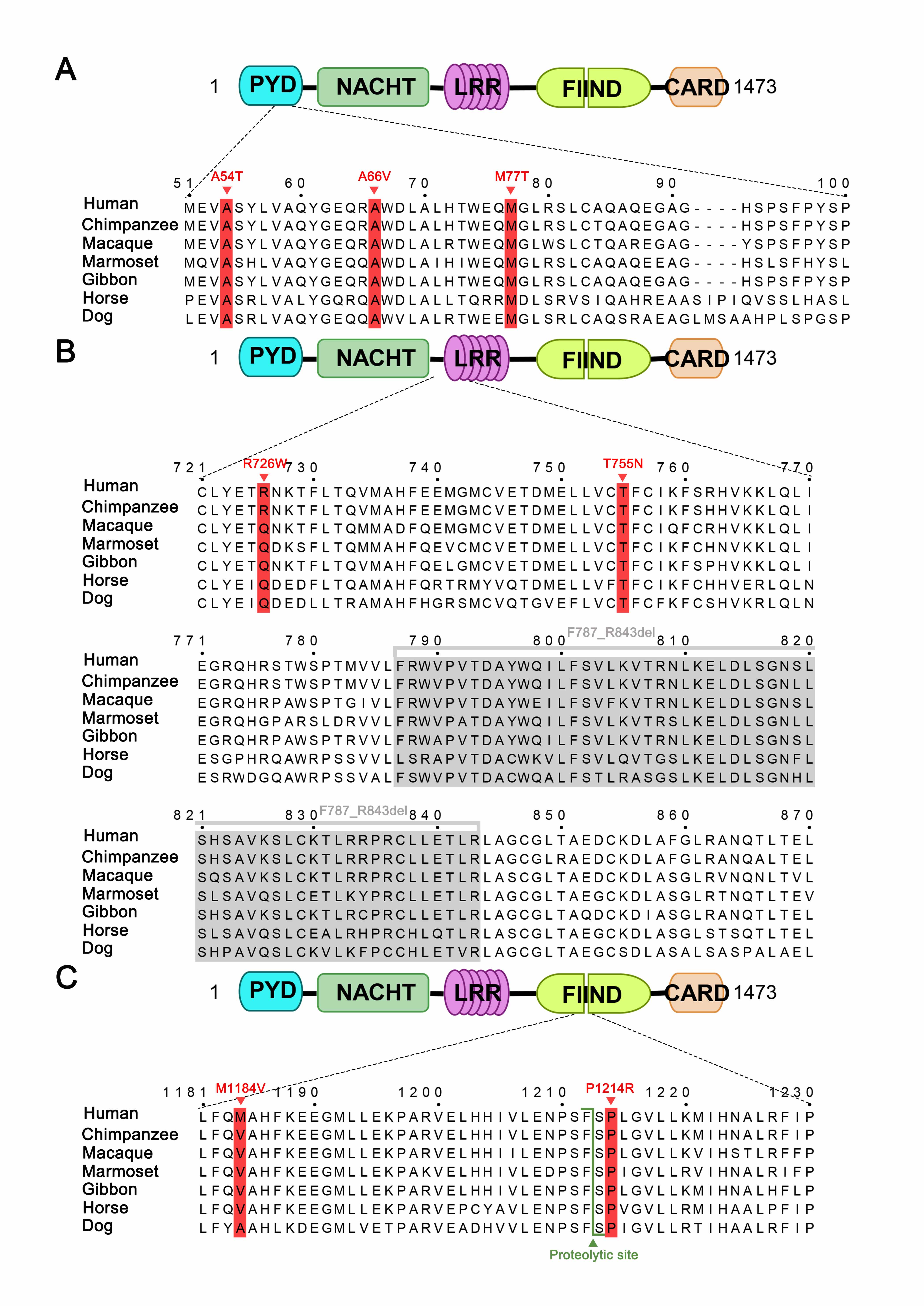

Supplement: Supplementary Figure 6 — Sequence alignment analysis of NLRP1 from indicated species. (A) Sequence alignment analysis of PYD domain of NLRP1 from indicated species. Disease-causing mutations are highlighted in red. (B) Sequence alignment analysis of interdomain linker between NLRP1NACHT domain and NLRP1LRR domain from indicated species. Disease-causing mutations are highlighted in red or marked with gray shade. (C) Sequence alignment analysis of interdomain sequences between NLRP1ZU5 subdomain and NLRP1UPA subdomain from indicated species. Disease-causing mutations are highlighted in red and proteolytic site is marked with green triangle arrow. [file Image_6.jpeg]
