# Supplementary material for: The CARD8 T60 variant associates with NLRP1 and negatively regulates its activation
Source: Front Immunol. 2022 Nov 8;13:1047922. doi: 10.3389/fimmu.2022.1047922 (PMC9679424; doi:10.3389/fimmu.2022.1047922)
Supplement: Supplementary file 7 [file DataSheet_1.pdf]

Vectors and plasmids	Abbreviation	Mutated amino acid sites
pcDNA3.1 Strep-NLRP1 UPA-CARD	A1-1385	Y1385H
pcDNA3.1 Strep-NLRP1 UPA-CARD	A1-1409/1410	S1409N, Q1410E
pcDNA3.1 Strep-NLRP1 UPA-CARD	A1-1415/1417	R1415A, L1417R
pcDNA3.1 Strep-NLRP1 UPA-CARD	A1-1423/1425	P1423Q, Q1425K
pcDNA3.1 Strep-NLRP1 UPA-CARD	A1-1434	Q1434R
pcDNA3.1 Strep-NLRP1 UPA-CARD	A1-1437/1439	D1437G, K1439A
pcDNA3.1 Strep-NLRP1 UPA-CARD	B1-F4M	Y1385H,R1415A,L1417R,P1423Q,Q1425K,Q1434R
pcDNA3.1 Strep-NLRP1 UPA-CARD	B1-F4M2	S1409N,Q1410E,R1415A,L1417R,P1423Q,Q1425K,D1437G,K1439A
pcDNA3.1 Strep-NLRP1 UPA-CARD	B1-F5M	Y1385H,S1409N,Q1410E,R1415A,L1417R,P1423Q,Q1425K,Q1434R
pcDNA3.1 Strep-NLRP1 UPA-CARD	B1-F6M	Y1385H,S1409N,Q1410E,R1415A,L1417R,P1423Q,Q1425K,Q1434R,D1437G, K1439A
pcDNA3.1 Strep-NLRP1 UPA-CARD	B1-F6M-1397-1399	Y1385H,E1397D,V1398P,V1399L,S1409N, Q1410E, R1415A,L1417R,P1423Q,Q1425K,Q1434R,D1437G,K1439A
pcDNA3.1 Strep-NLRP1 UPA-CARD	B1-F6M-1422-1425	Y1385H,S1409N,Q1410E,R1415A,L1417R,R1422N,P1423Q,S1424D,Q1425K, Q1434R,D1437G,K1439A
pcDNA3.1 Strep-NLRP1 UPA-CARD	B1-F6M-1443-1444	Y1385H,S1409N,Q1410E,R1415A, L1417R,P1423Q,Q1425K,Q1434R,D1437G,K1439A,G1443L,L1444F
pcDNA3.1 Strep-NLRP1 UPA-CARD	C1-F6M-S2M	Y1385H, E1397D,V1398P, V1399L,S1409N,Q1410E,R1415A,L1417R,R1422N,P1423Q,S1424D,Q1425K,Q1434R,D1437G,K1439A
pcDNA3.1 Strep-NLRP1 UPA-CARD	C1-F6M-S3M	Y1385H,E1397D,V1398P, V1399L,S1409N, Q1410E,R1415A,L1417R,R1422N,P1423Q,S1424D,Q1425K,Q1434R,D1437G,K1439A,G1443L,L1444F
