# Supplementary material for: The CARD8 T60 variant associates with NLRP1 and negatively regulates its activation
Source: Front Immunol. 2022 Nov 8;13:1047922. doi: 10.3389/fimmu.2022.1047922 (PMC9679424; doi:10.3389/fimmu.2022.1047922)
Supplement: Supplementary file 8 [file DataSheet_2.pdf]

Table S2 X-ray Data Collection and Structure Refinement of NLRP1^{LRR}

	NLRP1^{LRR}
**Data Collection**	
Space group	R32
Unit cell (a, b, c) (Å)	141.48, 141.48, 294.00
(α, β, γ) (°)	90, 90, 120
Wavelength (Å)	0.9000
Wilson B-factor (Å^2)	59.55
Resolution (last shell) (Å)	33.76 - 2.45 (2.53 - 2.45)
No of reflections (total/unique)	223505 (22677)
Completeness (%)	98.9 (99.1)
Average multiplicity	5.4 (5.6)
Mean $I/\sigma(I)$	18.99 (1.90)
R_{meas} (%)	6.4 (93.9)
R_{merge} (%)	5.716 (95.33)
R_{pim} (%)	2.682 (43.63)
$CC_{1/2}$	0.999 (0.678)
**Refinement**	
Resolution (Å)	50 - 2.45
Reflections used in refinement	41415
No. of protein atoms /*B*-factor (Å^2)	6210/70.45
No. of heteroatoms/*B*-factor (Å^2)	19/84.0
RMSD bond lengths (Å)	0.009
RMSD bond angles (°)	1.07
R_{work} (%)†	21.25
R_{free} (%)‡	25.93

Ramachandran plot favored/disallowed (%)[**]	95/0
PDB code	5Y3S

¶ $R_{merge} = \Sigma_h \Sigma_i |I_i(h) - <I(h)> | / \Sigma_h \Sigma_i I_i(h)$, where $I_i(h)$ and $<I(h)>$ are the ith and mean measurement of the intensity of reflection *h*.

¶ $R_{meas} = \Sigma_h (n/n-1)^{1/2} \Sigma_i |I_i(h) - <I(h)> | / \Sigma_h \Sigma_i I_i(h)$, where $I_i(h)$ and $<I(h)>$ are the *i*th and mean measurement of the intensity of reflection *h*.

† $R_{work} = \Sigma_h ||F_{obs}(h)|-|F_{calc}(h)|| / \Sigma_h |F_{obs}(h)|$, where $F_{obs}(h)$ and $F_{calc}(h)$ are the observed and calculated structure factors, respectively. No I/σ cutoff was applied.

‡ R_{free} is the R value obtained for a test set of reflections consisting of a randomly selected 5% subset of the data set excluded from refinement.

** Values from Molprobity server (http://molprobity.biochem.duke.edu/).
